# A stretchable wireless wearable bioelectronic system for multiplexed monitoring and combination treatment of infected chronic wounds

**DOI:** 10.1126/sciadv.adf7388

**Published:** 2023-03-24

**Authors:** Ehsan Shirzaei Sani, Changhao Xu, Canran Wang, Yu Song, Jihong Min, Jiaobing Tu, Samuel A. Solomon, Jiahong Li, Jaminelli L. Banks, David G. Armstrong, Wei Gao

**Affiliations:** ^1^ Andrew and Peggy Cherng Department of Medical Engineering, Division of Engineering and Applied Science, California Institute of Technology, Pasadena, CA 91125, USA.; ^2^ Keck School of Medicine, University of Southern California, Los Angeles, CA 90033, USA.

## Abstract

Chronic nonhealing wounds are one of the major and rapidly growing clinical complications all over the world. Current therapies frequently require emergent surgical interventions, while abuse and misapplication of therapeutic drugs often lead to an increased morbidity and mortality rate. Here, we introduce a wearable bioelectronic system that wirelessly and continuously monitors the physiological conditions of the wound bed via a custom-developed multiplexed multimodal electrochemical biosensor array and performs noninvasive combination therapy through controlled anti-inflammatory antimicrobial treatment and electrically stimulated tissue regeneration. The wearable patch is fully biocompatible, mechanically flexible, stretchable, and can conformally adhere to the skin wound throughout the entire healing process. Real-time metabolic and inflammatory monitoring in a series of preclinical in vivo experiments showed high accuracy and electrochemical stability of the wearable patch for multiplexed spatial and temporal wound biomarker analysis. The combination therapy enabled substantially accelerated cutaneous chronic wound healing in a rodent model.

## Graphical abstract

**Figure:**
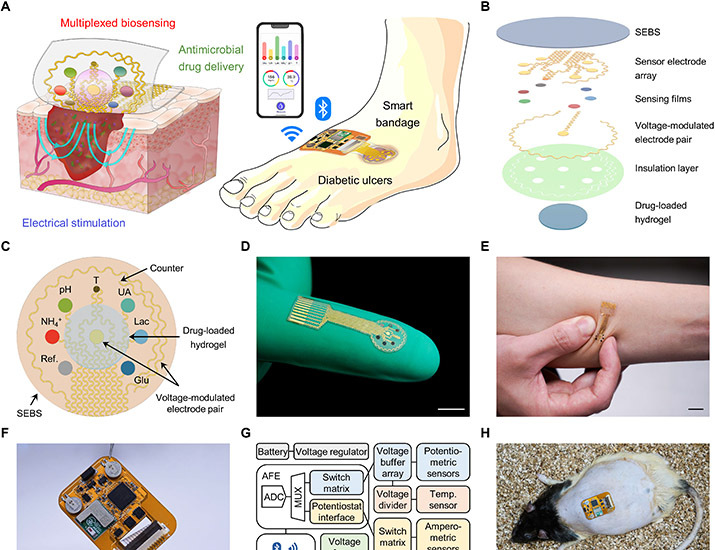

## Teaser

A soft smart bandage is capable of multiplexed metabolic monitoring, antimicrobial treatment, and electrical stimulation.

## INTRODUCTION

Chronic wounds are characterized by impaired or stagnant healing, prolonged and uncontrolled inflammation, as well as compromised extracellular matrix (ECM) function (*1*–*3*). Over 6.7 million people in the United States alone suffer from chronic nonhealing wounds including diabetic ulcers, nonhealing surgical wounds, burns, and venous-related ulcerations (*4*, *5*), causing a staggering financial burden of over $25 billion per year on the health care system (*6*). Chronic wound healing is a highly complex biological process consisting of four integrated and overlapping phases: hemostasis, inflammation, proliferation, and remodeling (*1*–*3*). Current therapies including skin grafts, skin substitutes, negative pressure wound therapy, and others can be beneficial but frequently require procedures or surgical intervention (*7*). Microbial infection at the wound site can severely prolong the healing process and lead to necrosis, sepsis, and even death (*3*). Both topical and systemic antibiotics are increasingly prescribed to patients suffering from chronic nonhealing wounds, but the overuse, abuse, and misapplication of antibiotics often lead to an escalating drug resistance in bacteria, causing a drastic increase in morbidity and mortality rates (*8*). As an alternative therapeutic approach, electrical stimulation has shown to have a substantial effect on the wound healing process, including stimulating fibroblast proliferation and differentiation into myofibroblasts and collagen formation, keratinocyte migration, angiogenesis, and attracting macrophages (*9*, *10*). However, currently reported electrical stimulation devices usually require bulky equipment and wire connections, making them highly challenging for practical clinical use. More effective, fully controllable, and easy-to-implement therapies are critically needed for personalized treatment of chronic wounds with minimal side effects.

At each stage of healing process, the chemical composition of the wound exudate changes substantially, indicating the stage of healing and even the presence of an infection (*11*–*13*). For example, increased temperature is associated with bacterial infection, and changes in temperature can provide information on various factors relevant to healing, inflammation, and oxygenation in the wound bed; acidity (pH) indicates a healing state with balanced protease activities and effective ECM remodeling, moreover, elevated pH in wound environment can be a sign of infection; elevated uric acid (UA) indicates wound severity with excessive reactive oxygen species and inflammation and shows immune system responding to inflammatory cytokines (*14*); lactate and ammonium are crucial markers for soft-tissue infection diagnosis and angiogenesis in diabetic foot ulcers (*15*); wound exudate glucose has a strong correlation with blood glucose and bacterial activities (*16*), providing crucial therapeutical guidance for clinical diabetic wound treatment.

Recent advances in digital health and flexible electronics have transformed conventional medicine into remote at-home health care (*17*–*23*). Wearable biosensors could allow real-time and continuous monitoring of physical vital signs and physiological biomarkers in various biofluids such as sweat, saliva, and interstitial fluids (*18*–*21*, *24*–*30*). In general, an ideal wound dressing should provide a moist wound environment, offer protection from secondary infections, remove wound exudate, and promote tissue regeneration. Despite the promising prospects opened by the wearable technologies (*31*–*37*), major challenges exist to realize their full potential toward practical chronic wound management applications: the chronic nonhealing wounds pose high requirement on the flexibility, breathability, and biocompatibility of the wearable devices to protect the wound bed from bacterial infiltrations and infection and modulate wound exudate level; the complex wound exudate matrix could substantially affect the biosensor performance, and thus, there are few reports on prolonged evaluation of biosensors in vivo (*13*, *31*); personalized wound management demands both effective wound therapy and close monitoring of crucial wound healing biomarkers in the wound exudate; the absence of miniaturized user-interactive fully integrated closed-loop wearable systems and the evaluation of such systems in vivo impede their practical use.

**Fig. 1.**
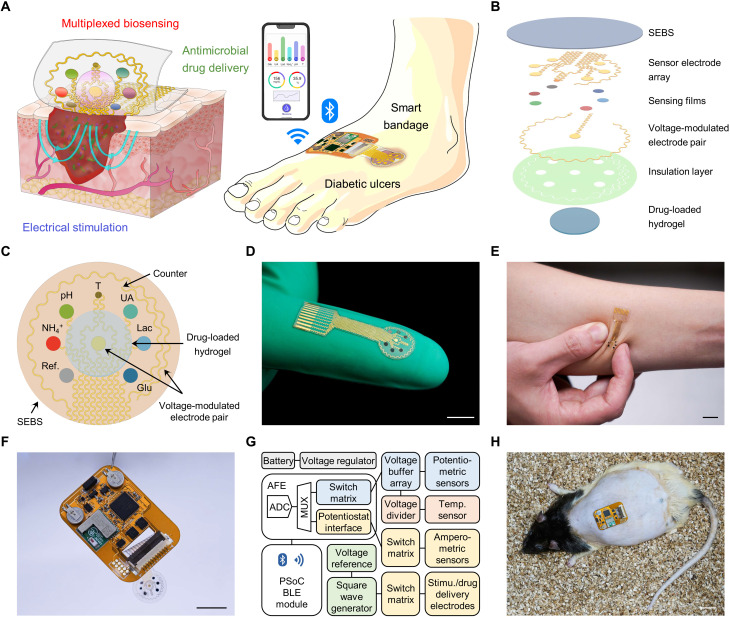
A wireless stretchable wearable bioelectronic system for multiplexed monitoring and treatment of chronic wounds. (**A**) Schematic of a soft wearable patch on an infected chronic nonhealing wound on a diabetic foot. (**B**) Schematic of layer assembly of the wearable patch, showing the soft and stretchable poly[styrene-*b*-(ethylene-*co*-butylene)-*b*-styrene] (SEBS) substrate, the custom-engineered electrochemical biosensor array, a pair of voltage-modulated electrodes for controlled drug release and electrical stimulation, and an anti-inflammatory and antimicrobial drug-loaded electroactive hydrogel layer. (**C**) Schematic layout of the smart patch consisting of a temperature (T) sensor, pH, ammonium (NH_4_^+^), glucose (Glu), lactate (Lac), and UA sensing electrodes, reference (Ref) and counter electrodes, and a pair of voltage-modulated electrodes for controlled drug release and electrical stimulation. (**D** and **E**) Photographs of the fingertip-sized stretchable and flexible wearable patch. Scale bars, 1 cm. (**F** and **G**) Schematic diagram (F) and photograph (G) of the fully integrated miniaturized wireless wearable patch. Scale bar, 1 cm. ADC, analog to digital converter; AFE, analog front end; PSoC, programmable system on chip; MUX, multiplexer; BLE, Bluetooth Low Energy. (**H**) Photograph of a fully integrated wearable patch on a diabetic rat with an open wound. Scale bar, 2 cm.

To address these challenges, here, we introduce a fully integrated wireless wearable bioelectronic system that effectively monitors the physiological conditions of the wound bed via multiplexed and multimodal wound biomarker analysis and performs combination therapy through electro-responsive controlled drug delivery for anti-inflammatory antimicrobial treatment and exogenous electrical stimulation for tissue regeneration (Fig. 1, A and B). The wearable patch is mechanically flexible, stretchable, and can conformally adhere to the skin wound throughout the entire wound healing process, preventing any undesired discomfort or skin irritation. Because of the wound’s complex pathophysiological environment, compared to previously reported single-analyte sensing, multiplexing analysis of wound exudate biomarkers can provide more comprehensive and personalized information for effective chronic wound management. In this regard, a panel of wound biomarkers including temperature, pH, ammonium, glucose, lactate, and UA were chosen on the basis of their importance in reflecting the infection, metabolic, and inflammatory status of the chronic wounds. Real-time selective monitoring of these biomarkers in complex wound exudate could be realized in situ using custom-engineered electrochemical biosensor arrays (Fig. 1C). The wearable system’s capabilities of multiplexed monitoring, biomarker mapping, and combination therapy were evaluated in vivo over prolonged periods of time in rodent models with infected diabetic wounds. The multiplexed biomarker information collected by the wearable patch revealed both spatial and temporal changes in the microenvironment as well as inflammatory status of the infected wound during different healing stages. In addition, the combination of electrically modulated antibiotic delivery with electrical stimulation on the wearable technology enabled substantially accelerated chronic wound closure.

## RESULTS

### Design of the fully integrated stretchable wearable bioelectronic system

The disposable wearable patch consists of a multimodal biosensor array for simultaneous and multiplexed electrochemical sensing of wound exudate biomarkers, a stimulus-responsive electroactive hydrogel loaded with a dual-function anti-inflammatory and antimicrobial peptide (AMP), as well as a pair of voltage-modulated electrodes for controlled drug release and electrical stimulation (Fig. 1, B and C). The multiplexed sensor array patch is fabricated via standard microfabrication protocols on a sacrificial layer of copper followed by transfer printing onto a poly[styrene-*b*-(ethylene-*co*-butylene)-*b*-styrene] (SEBS) thermoplastic elastomer substrate (figs. S1 and S2). The serpentine-like design of electronic interconnects, and the highly elastic nature of SEBS enables high stretchability and resilience of the sensor patch against undesirable physical deformations (Fig. 1, D and E). The flexible bandage seamlessly interfaces with a flexible printed circuit board (FPCB) for electrochemical sensor data acquisition, wireless communication, and programmed voltage modulation for controlled drug delivery and electrical stimulation (Fig. 1, F to H, and figs. S3 to S5). The wireless wearable device can be attached to the wound area with firm adhesion, allowing the animals to move freely over a prolonged period (movie S1 and figs. S6 and S7).

### Design and characterization of the soft sensor array for multiplexed biomarker analysis

**Fig. 2.**
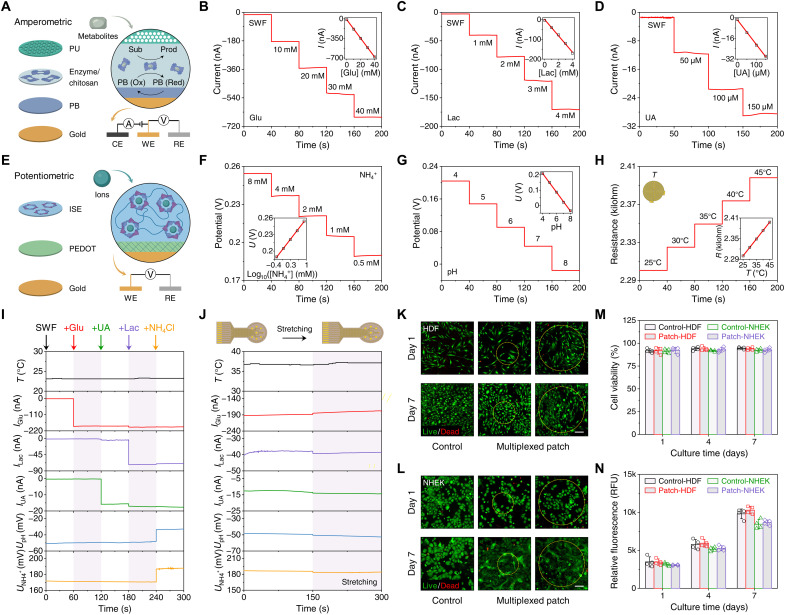
Design and characterization of the sensor array for multiplexed wound analysis. (**A** to **D**) Schematic (A) and chronoamperometric responses of the enzymatic glucose (B), lactate (C), and UA (D) sensors in SWF. Insets in (B) to (D), the calibration plots with a linear fit. PB, Prussian blue; Sub, substrate; Prod, product; CE, counter electrode; WE, working electrode; RE, reference electrode; *I*, current. (**E** and **F**) Schematic (E) and potentiometric response (F) of an NH_4_^+^ sensor in SWF. Insets in (F), the calibration plot with a linear fit. ISE, ion-selective electrode; PEDOT, poly(3,4-ethylenedioxythiophene); *U*, potential. (**G**) Potentiometric response of a polyaniline-based pH sensor in McIlvaine buffer. Insets, the calibration plot with a linear fit. (**H**) Resistive response of an Au microwire–based temperature sensor under temperature changes in physiologically relevant range in SWF. Insets, schematic of a temperature sensor and the calibration plot with a linear fit. All error bars in (A) to (H) represent the SD from three sensors. (**I**) Selectivity study of the multiplexed sensor array in SWF. Ten millimolar glucose, 50 μM UA, 1 mM lactate, and 1 mM NH_4_^+^ were added sequentially to the SWF. (**J**) Responses of the multiplexed sensor array before and during mechanical stretching (15%) in SWF (pH 8) containing 10 mM glucose, 50 μM UA, 1 mM lactate, and 0.25 mM NH_4_^+^. (**K** and **L**) Representative live (green)/dead (red) images of human dermal fibroblasts (HDFs) (K) and normal human epidermal keratinocytes (NHEKs) (L) cells seeded on the multiplexed sensor array and in PBS (control) after 1-day and 7-day culture. Scale bars, 200 μm. (**M** and **N**) Quantitative analysis of cell viability images (M) and cell metabolic activity (N) over a 7-day period after culture. RFUs, relative fluorescence units. Error bars represent the SD (*n* = 4).

The array of flexible biosensors was custom developed to allow real-time multiplexed monitoring of the biomarkers in complex wound exudate. The continuous and selective measurement of glucose, lactate, and UA is based on amperometric enzymatic electrodes with glucose oxidase, lactate oxidase, and uricase immobilized in a highly permeable, adhesive, and biocompatible chitosan film, respectively (Fig. 2A). Electrodeposited Prussian blue (PB) serves as the electron-transfer redox mediator for the enzymatic reaction, which allows the biosensors to operate at a low potential (~0.0 V) to minimize the interferences of oxygen and other electroactive molecules. Because of the complex and heterogeneous composition of wound fluid (e.g., high protein levels, local and migrated cells, and exogenous factors such as bacteria) (*13*), previously reported enzymatic sensors suffer from severe matrix effects and fail to accurately measure the target metabolite levels in untreated wound fluid (figs. S8 and S9 and note S1). Moreover, high levels of metabolites in diabetic wound fluid, especially glucose (up to 50 mM), pose another major challenge to obtain linear sensor response in the physiological concentration ranges. To address these issues and achieve accurate wound fluid metabolic monitoring, increase sensor range, and minimize biofouling effects, we explored the use of an outer porous membrane that serves as a diffusion limiting layer to protect the enzyme, tune response, increase operational stability, as well as enhance the linearity and sensitivity magnitude of the sensor. We fabricated our enzymatic glucose oxidase/chitosan/single-walled carbon nanotubes (GOx/CS/MWCNT) glucose sensor with additional porous membrane coatings including CS, poly(ethylene glycol) diglycidyl ether (PEGDGE), Nafion, and polyurethane (PU) (fig. S9). As expected, the addition of diffusion layers indeed improves the sensor’s linear range in simulated wound fluid (SWF). However, CS-, PEGDGE-, and Nafion-coated sensors did not show reliable responses in wound fluid upon the addition of glucose. The PU-based enzymatic sensors showed the highest linearity over the wide physiological concentration range as well as high reproducibility in complex wound fluid matrix (fig. S10). The amperometric current signals generated from the PU-coated enzymatic glucose, lactate, and UA sensors are proportional to the physiologically relevant concentrations of the corresponding metabolites in SWF with sensitivities of 16.34, 41.44, and 189.60 nA mM^−1^, respectively (Fig. 2, B to D). Continuous monitoring of ammonium is based on a potentiometric ion-selective electrode where the binding of ammonium with its ionophore results in an electrode potential log-linearly corresponding to the target ion concentration with a sensitivity of 59.7 mV decade^−1^ (Fig. 2, E and F). Similarly, the pH sensor uses an electrodeposited polyaniline film as the pH-sensitive membrane and shows a sensitivity of 59.7 mV per pH (Fig. 2G). For all chemical sensors, a polyvinyl butyral (PVB)–coated Ag/AgCl electrode was used as the reference electrode that provides a stable voltage independent of the variations of wound fluid compositions (*24*). A gold microwire-based resistive temperature sensor is integrated as part of the sensor array and shows a sensitivity of approximately 0.21% °C^−1^ in the physiological temperature range of 25° to 45°C (Fig. 2H).

Considering that other electrolytes and metabolites present in wound fluid may negatively affect the sensor outputs, we examined the selectivity of the sensor array consisting of all six sensors. As illustrated in Fig. 2I, the addition of nontarget electrolytes and metabolites did not trigger any substantial interference to the sensor response. Moreover, all biosensors showed high selectivity over nonspecific compounds when evaluated in SWF (fig. S11). It should be noted that while temperature has negligible effects on the potentiometric sensors, it substantiallyinfluences the performance of the enzymatic sensors due to the temperature-dependent enzyme activities (fig. S12). Moreover, our data show that the medium pH could also affect the performance of enzymatic sensors (fig. S13). With pH and temperature sensors integrated into the wearable patch, we are able to perform real-time adjustments and calibration of the enzymatic biosensors based on temperature and pH variations to realize accurate wound metabolite analysis.

Owing to the soft SEBS substrate and the serpentine-like design of electronic interconnects, the wound patch showed excellent mechanical flexibility and stretchability, which are essential to maintaining good contact with the skin in vivo during the chronic wound healing process. Negligible alterations in the sensor responses before and under unidirectional tensile stretching (Fig. 2I) and after repetitive mechanical bending (fig. S14) were observed, indicating highly consistent sensor performance under various physical deformations.

As the sensor patch is designed for long-term in vivo use, its cytocompatibility and biocompatibility are of great importance. Cell viability and metabolic activity of the cells seeded on a multiplexed sensor array were analyzed using a commercial live/dead kit and PrestoBlue assay, respectively (Fig. 2, K to N, and fig. S15). The high cell viabilities shown in the representative live/dead staining images of human dermal fibroblasts (HDFs) and normal human epidermal keratinocytes (NHEK) cells (Fig. 2, K to M, and fig. S15), along with the consistently increased cell metabolic activities (Fig. 2N) over multiday culture periods, indicate the high cytocompatibility of the soft sensor patch.

### Characterization of the therapeutic capabilities of the wearable patch in vitro

**Fig. 3.**
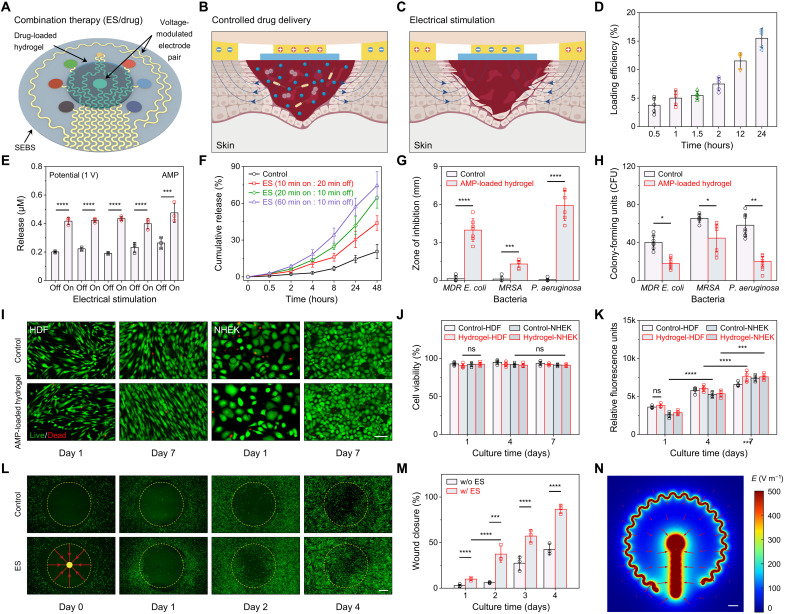
Characterization of the therapeutic capabilities of the wearable patch in vitro. (**A** to **C**) Schematic illustration of the therapeutic modules of the wearable patch (A) and the working mechanisms of the controlled drug delivery for antimicrobial treatment (B) and electrical stimulation for tissue regeneration (C). (**D**) Loading efficiency of dual-functional TCP-25 anti-inflammatory and AMP into CS electroactive hydrogel after 0.5- to 24-hour incubation. (**E**) Release amount of AMP from the hydrogel under programmed on-off electrical voltage (1 V, 10 min each step). (**F**) Long-term cumulative release of the AMP under programmed electrical modulation. (**G** and **H**) In vitro antimicrobial tests including zone of inhibition (G) and colony forming units (H) assays for electroactive hydrogels with and without TCP-25 AMP against multidrug-resistant *Escherichia coli* (MDR *E. coli*), *P. aeruginosa*, and methicillin-resistant *Staphylococcus aureus* (MRSA). (**I** to **K**) In vitro cytocompatibility assessment of TCP-25–loaded electroactive hydrogels using live/dead staining (I) and quantification of cell viability (J) and metabolic activity (K) for HDF and NHEK cells cultured in the presence of hydrogels. Scale bar, 100 μm. (**L** and **M**) Fluorescence images (L) and quantitative wound closure analysis (M) to evaluate the wearable patch’s therapeutic capability via electrical stimulation using an in vitro circular wound healing assay created by HDF cells. ES, electrical stimulation. A pulsed voltage was applied for electrical stimulation (1 V at 50 Hz, 0.01 s voltage on for each cycle). Scale bar, 500 μm. (**N**) Numerical simulation of the electrical field generated by the custom-designed electrical stimulation electrodes during operation. *E*, electrical field. Scale bar, 500 μm. Error bars represent the SD (*P < 0.05, **P < 0.01, ***P < 0.001, and ****P < 0.0001; *n* ≥ 3). ns, not significant.

In addition to the multiplexed and multimodal biosensing, the wearable patch is able to perform combination treatment of chronic wounds through drug release from an electroactive hydrogel layer and electrical stimulation under an exogenic electric field, both controlled by a pair of voltage-modulated electrodes (Fig. 3, A to C). The electroactive hydrogel consists of chondroitin 4-sulfate (CS), a sulfated glycosaminoglycan composed of units of glucosamine, cross-linked with 1,4-butanediol diglycidyl ether (fig. S16). Because of the shear-thinning behavior of the prepolymer solution, the hydrogel can be precisely fabricated via three-dimensional (3D) printing (fig. S17). The negatively charged CS hydrogel is an ideal choice for loading and controlled release of positively charged large biological drug molecules based on an electrically modulated “on/off” drug release mechanism (Fig. 3B). Here, an AMP, thrombin-derived c-terminal peptide-25 (TCP-25) (*38*), was loaded within the CS hydrogel network through the electrostatic interactions with the polymer backbone, with up to 15% loading efficiency (Fig. 3D). The highly porous hydrogel network under equilibrium swelling could further enhance the drug loading efficiency (fig. S18). Under an applied positive voltage, the electroactive hydrogels will be rapidly protonated, resulting in anisotropic and microscopic contraction followed by syneresis/expelling of water from the gel (*39*) and consequently allowing a controlled release of the TCP-25 AMP (Fig. 3, E and F, and figs. S19 and S20). In addition, the electrical field will also facilitate the diffusion of positively charged AMP out of the stimuli-sensitive CS hydrogel toward the cathode due to electrophoretic flow (*40*).

The antimicrobial activity of the TCP-25 AMP–loaded hydrogel was evaluated against Gram-positive methicillin-resistant *Staphylococcus aureus* (MRSA) and *Pseudomonas aeruginosa*, and Gram-negative multidrug-resistant *Escherichia coli* (MDR *E. coli*) and *Staphylococcus epidermidis*, the most common pathogenic bacteria associated with microbial colonization of chronic nonhealing wounds (Fig. 3, G and H, and fig. S21). The zone of inhibition assay indicates the susceptibility of the MDR *E. coli*, *P. aeruginosa*, and MRSA toward TCP-25 AMP (Fig. 3G), while the standard colony-forming units (CFU) showed that the drug-loaded hydrogel was effectively protected from all pathogenic colonization (Fig. 3H). For cells cultured on antimicrobial peptide (AMP)-loaded hydrogels, the viability of HDF and NHEK cells remained >90%, and their metabolic consistently increased during the 7-day culture (Fig. 3, I to K, and fig. S22), indicating that the TCP-25–loaded gels are highly cytocompatible and support cell proliferation.

The wearable patch’s therapeutic capability toward enhanced tissue regeneration via electrical stimulation was assessed using an in vitro wound healing assay (Fig. 3, L and M, and fig. S23). The model wound treated with electrical stimulation showed substantially faster and more consistent migration of HDF cells toward the wound area for four consequent days after wounding as compared to the control group without electrical stimulation (Fig. 3L). Quantitative analysis of the model wound closure indicates higher wound closure rates in the wounds treated with electrical stimulation (Fig. 3M). The enhanced tissue regeneration is attributed to the directional electrical field generated from our custom-designed electrical stimulation electrodes (Fig. 3N), which plays a crucial role in cell behavior modulation including cell-cell junctions, cell division orientation, and cell migration trajectories (galvanotaxis or electrotaxis) (*41*–*43*). The electrical potential was applied directly to a pair of insulated electrodes to generate electrical field for electrical stimulation. It should also be noted that continuous electrical stimulation did not cause substantial temperature increase (fig. S24).

### Evaluation of the wearable patch in vivo for multiplexed wound biomarker monitoring

**Fig. 4.**
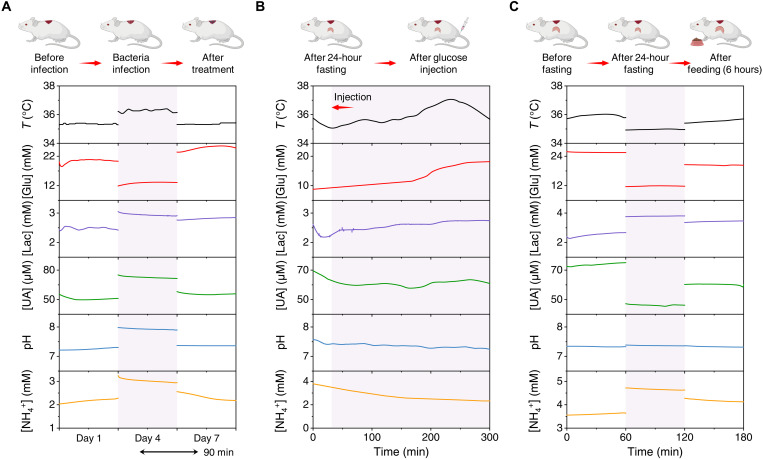
In vivo evaluation of the wearable patch for multiplexed wound biomarker monitoring in a wound model in diabetic mice. (**A**) In vivo multiplexed analysis of the chemical composition of wound fluid using a wearable patch in an infected excisional wound model in a diabetic mouse. Infection and treatment were performed after the sensor recording on days 1 and 4, respectively. (**B**) In vivo continuous and multiplexed evaluation of wound parameters in a 24-hour fasted mouse before and after glucose administration via tail vein. (**C**) In vivo assessment of metabolic changes in wound microenvironment in response to fasting and food feeding in a diabetic mouse.

To validate the capability and efficacy of our wearable patch, in vivo preclinical evaluations are essential. In this regard, the in vivo biocompatibility of the wearable patch was assessed. The immunohistofluorescent staining of subcutaneously implanted hydrogel and electrodes in rats showed negligible signs of leukocyte (CD3) and macrophage (CD68) antigens after 56 days, indicating the high biocompatibility of the wearable patch (fig. S25). All custom-developed biosensors on the wearable patch displayed consistent sensitivity during a 6-hour continuous measurement in SWF, indicating the high electrochemical stability of the sensors for wound analysis (fig. S26). In vivo multiplexed sensing study was then performed using an infected excisional wound model in diabetic mice. The wound fluid composition was assessed by the wearable patch before infection (day 1), after infection (day 4), and after treatment (day 7) (Fig. 4A). Substantially elevated UA, temperature, pH, lactate, and ammonium levels were observed as compared to those before infection. The increase in temperature can be potentially linked to inflammation (*44*). The elevated levels of UA after infection can be due to up-regulation of xanthine oxidase, a component of the innate immune system responding to inflammatory cytokines in chronic ulcers that plays a key role in purine metabolism to produce UA (*45*). pH, lactate, and ammonium are all acidity related, and their elevation during the bacteria infection has also been widely reported (*46*). In contrast, the glucose level in infected wound fluid showed >35% decrease after infection, attributing to the increased glucose consumption of bacteria activities (*16*). Upon wound treatment, the temperature, pH, lactate, UA, and ammonium decreased toward the levels before the infection, while the glucose level increased significantly after treatment, indicating the successful bacterial elimination (Fig. 4A).

Considering that dietary intake may have major impact on the composition of diabetic wound fluid, we evaluated the metabolic changes in wound fluid in response to tail vein glucose administration (Fig. 4B) and food feeding (Fig. 4C). Glucose administration via tail vein into the 24-hour fasted mice sparked ~10 mM increase in the blood glucose level. The in vivo sensor readings from the wearable patch were recorded from 30 min before injection and continued until 270 min after injection (Fig. 4B). The glucose level in wound fluid showed a gradual increase throughout the 4 hours after injection, indicating a protracted delay with respect to blood glucose. A similar trend was observed for temperature values, attributing to an increased metabolic rate to facilitate digestion. No apparent change in UA level after injection was detected because of the absence of purine intake in the glucose administration. For the food feeding study, the wearable patch was tested before fasting, after 24-hour fasting, and 6 hours after feeding (Fig. 4C). The lactate and ammonium levels increased substantially after fasting, while glucose and UA levels decreased after fasting, consistent with the trend of observed blood level changes (*47*). In the meantime, temperature decreased due to the fasting-induced hypothermia (*48*). As expected, 6 hours after feeding, the glucose and UA levels increased from 11.9 to 20.3 mM and from 45.9 to 60.3 μM, respectively. These results indicate that wearable patch-enabled wound fluid analysis could be a promising approach to realize continuous and personalized metabolic monitoring.

### Spatial and temporal monitoring of critical-sized wounds using the wearable patch

**Fig. 5.**
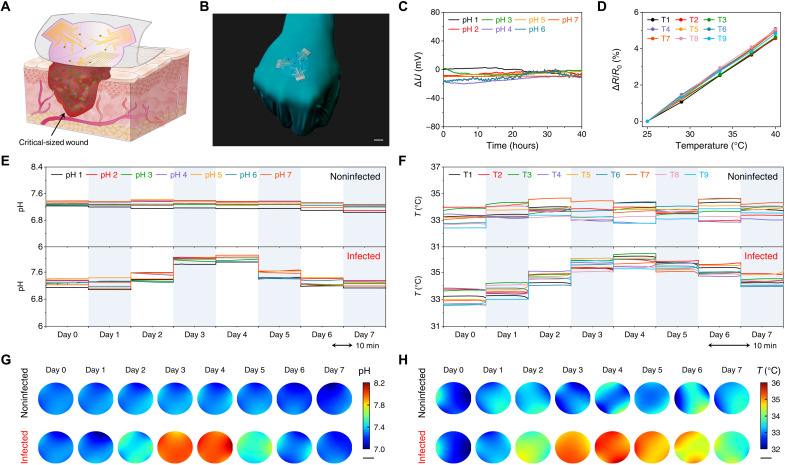
Spatial and temporal monitoring of critical-sized full-thickness infected wound defects in diabetic rats using the wearable patch. (**A** and **B**) Schematic (A) and photograph (B) of a soft sensor patch with pH and temperature sensor arrays designed for spatial and temporal monitoring of large and irregular wounds. Scale bar, 1 cm. (**C** and **D**) The characterization of pH (C) and temperature (D) sensor arrays on a wearable patch in SWF solutions. (**E** and **F**) Dynamic changes in pH (E) and temperature (F) values of each biosensor on a wearable patch for critical-sized noninfected and infected wounds. (**G** and **H**) The mapping of daily local pH (G) and temperature (H) sensor readings in the wound area for infected and noninfected wounds on each day over the 7-day study period.

The wearable patch is mass producible and readily reconfigurable for various wound care applications. In the case of large chronic ulcers, the wound parameters and microenvironment may vary from site to site, making localized monitoring crucial for optimized assessment and treatment of chronic wound infection. As a proof of concept, we demonstrate customized wearable patches for spatial mapping of physiological conditions of critical-sized wounds during the healing process. As illustrated in Fig. 5 (A and B), we could incorporate a sensor array containing seven pH sensors and nine temperature sensors onto our wearable platform for monitoring and mapping critical-sized full-thickness infected chronic wounds in diabetic rats. The pH and temperature sensor arrays showed high reproducibility and stability in SWF solutions before and after in vivo application (Fig. 5, C and D, and fig. S27).

On-body validation of the sensor array for spatial and temporal wound monitoring was conducted on critical-sized full-thickness wounds (35 mm in diameter) in Zucker diabetic fatty (ZDF) rats before infection, after infection, and after treatment. The dynamic changes in pH and temperature values for each biosensor on the wearable patch in noninfected and infected critical-sized wounds are illustrated in Fig. 5 (E and F). For noninfected wound studies, the pH and temperature values did not notably change over the 7-day period. However, for infected wound studies, the pH and temperature values increased daily upon applying a mixed infection (MRSA and *P. aeruginosa*) on day 1 and reached the peak value on days 3 and 4. Upon treatment on day 4, the pH and temperature values for each sensor decreased substantially and recovered toward the levels before the infection on day 7. The spatial mapping plots of pH (Fig. 5G) and temperature (Fig. 5H) in the chronic wound area on each day over the 7-day period were successfully generated on the basis of localized sensor readings. These results are in agreement with previous literature on the changes in the pH and temperature values during the healing progress (*46*). A wide variation was observed in both pH and temperature in different regions of the wound upon bacterial infiltration on day 2, showing a higher bacteria growth in the wound edges. The infected wound showed a more uniform pH and temperature at different regions 2 and 3 days after infection due to the formation of uniform biofilm. Upon treatment, the variations increased in the treated wounds on days 5 and 6, indicating the disruption and eventually elimination of the biofilm after treatment (Fig. 5, G and H).

### Evaluation of the therapeutic efficacy of the wearable patch in chronic wound healing in vivo

**Fig. 6.**
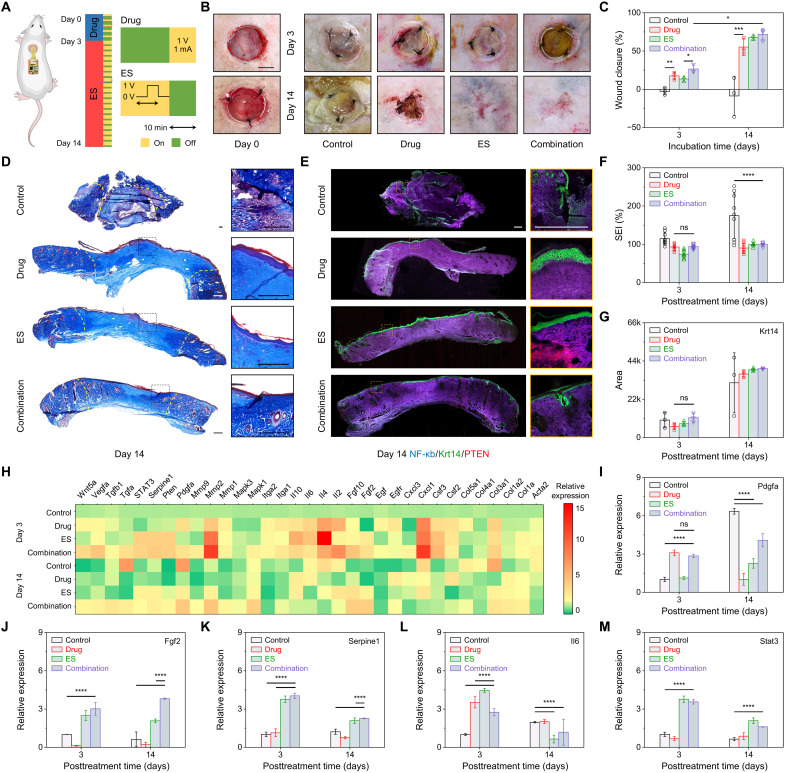
In vivo evaluation of wearable patch-facilitated chronic wound healing in full-thickness infected wounds in ZDF diabetic rats. (**A**) Schematic of the wearable patch on a diabetic wound and the working diagram of combination therapy. (**B** and **C**) Representative images (B) and quantitative analysis of wound closure (C) for the control wound and wounds treated with drug, ES, and combination therapy on days 3 and 14 after application. Scale bar, 500 μm. (**D**) Representative images of Masson’s trichrome (MTC)–stained sections of the full-thickness skin wounds after 14 days of combination treatment. Scale bars, 500 μm. (**E**) Representative immunofluorescent stained images for nuclear factor κB (NF-κB) (purple), keratin 14 (Krt14) (green), and phosphatase and tensin homolog (Pten) (red) 14 days after the treatment. Scale bars, 500 μm. (**F** and **G**) Quantitative analysis of scar elevation index (SEI) based on MTC images (F) and Krt14 marker based on immunofluorescent images (G). (**H**) Quantitative real-time polymerase chain reaction (qRT-PCR) analysis of a library of wound biomarkers for wound biopsies after 3 and 14 days of treatment. (**I** to **M**) Relative expression of Pdgfa (I), Fgf (J), Serpine1 (K), IL-6 (L), and Stat3 (M) genes after 3 and 14 days of treatment. Error bars represent the SD (*P < 0.05, **P < 0.01, ***P < 0.001, and ****P < 0.0001; *n* = 3).

The wearable patch-facilitated combination therapy and wound healing were evaluated in a splinted excisional wound model in ZDF diabetic rats (Fig. 6A). Four different groups were tested: negative control, drug release, electrical stimulation, and combination therapy. The drug treatment was primarily used to eliminate bacterial infections and regulate immune response in early stages of healing. The electrical stimulation was used to facilitate ion channel up-regulation and redistribution, resulting in accelerated cell migration and wound healing. The wearable patch’s high flexibility and stretchability provided intact and comfortable contact with the animal’s back curvature. Over a 14-day period, the animals were routinely weighed where infected rats showed a nonsubstantially lower body weight compared to noninfected animals (fig. S28), indicating that the study procedures did not have any substantial influence on the animals’ health. Moreover, the standard CFU on the mixed infection isolated from the wound beds 3 days after drug and combination therapy groups showed a significant reduction in bacterial growth as compared to control and electrical stimulation groups, suggesting effectiveness of the wearable patch in the elimination of pathogenic species from the wound (fig. S29). Substantially higher rates of wound closure were observed in the treated wounds as compared to the control untreated group, where the group that received combination therapy showed the highest wound closure rate, collagen deposition, and granulation tissue formation, suggesting the recovery of the wound toward the unwounded state (Fig. 6, B and C, and fig. S30). We also evaluated the use of the wearable system for multiplexed biosensing and the combination therapy on the same diabetic rats (fig. 31): Compared to the individual evaluation as shown in Figs. 5 and 6 (B and C), similar sensing results and therapeutic effects to the individual evaluation were observed: The continuous sensing data were obtained up to 8 days until the wound dried after therapy while the wound fully closed 14 days after surgery.

In addition, the histopathological analysis of sections of the full-thickness skin wounds via Masson’s trichrome (MTC) staining (Fig. 6D) and immunofluorescent staining (Fig. 6E) was performed. The MTC images showed a significantly higher collagen deposition and granulation tissue formation for treated groups compared to the control group on day 14 (Fig. 6D). Moreover, the control group on day 14 showed a significantly higher scar elevation index (SEI) of 175 ± 59%, indicating the formation of hypertrophic scars; in contrast, the SEI for combination treatment group was 100 ± 4%, showing uniform dermis repair after treatment (Fig. 6F). The combination therapy was able to accelerate the wound-induced hair follicle neogenesis with adjoining sebaceous glands within the wound bed (Fig. 6, D and E, insets) resembling structurally similar glands to those of the uninjured skin (*49*). The immunohistochemical analysis of keratin 14 (Krt14), a marker of undifferentiated keratinocytes, revealed a delayed re-epithelization in the control group (16%) as compared to the substantially accelerated re-epithelization in combination treatment group (99%) after 14 days (Fig. 6, E and G, and fig. S30). We further observed a significant growth in the expression of tumor suppressor phosphatase and tensin homolog (Pten), an indicator of higher electrotactic responses (*42*), among electrical stimulation and combination treatment groups as a direct result of electrical stimulation (Fig. 6E and fig. S30). A higher expression of nuclear factor κB (NF-κB) enhancer binding protein, a key signaling factor that promotes remodeling of cellular junctions, cell proliferation, and adhesion (*50*), was also observed in the combination therapy group on day 14, indicating a higher cutaneous wound healing (Fig. 6E and fig. S30).

We further studied the molecular mechanism behind the beneficial effects of our wearable patch’s combination treatment on wound healing using quantitative real-time polymerase chain reaction (qRT-PCR) analysis. A library of the most relevant genes associated with wound healing was screened. A substantially elevated expression of growth factors was confirmed in the combination-treated wounds (Fig. 6H, fig. S32, and note S2), while the control group on day 14 showed a reduced level of these growth factors due to compromised cutaneous wound healing that resulted in impaired re-epithelization and the formation of granulation tissue and ECM. Considering that platelet-derived growth factor subunit A (Pdgfa) plays crucial roles in stimulation of fibroblast proliferation (early function) and induces the myofibroblast phenotype (later function) (*51*), its elevation supports the higher rate of dermis and granulation tissue formation in the combination treatment group on day 14, while lower or delayed *Pdgfa *gene expression resulted in impaired wound healing in drug and electrical stimulation groups in the same period (Fig. 6I). The overexpression of *Pdgfa *gene in control group after 14 days might be due to the pathogenesis of hypertrophic scars and increased responsiveness of keloid fibroblasts to Pdgf (*52*). In addition, the higher expression of fibroblast growth factor (Fgf) genes can be due to higher rate of epidermis regeneration, renewed capillaries, and in cells infiltrating in the granulation tissue (Fig. 6J) (*53*). There was also a significant up-regulation of serine protease inhibitor clade E member 1 (Serpine1) genes in electrical stimulation and combination treatment groups as compared to the control and drug groups primarily due to the applied electrical field (Fig. 6K) (*54*). *Serpine1 *regulates the extent and location of matrix restructuring and collagen remodeling while facilitating cell motility and proliferation in the process of wound regeneration. Moreover, significant up-regulations of proinflammatory cytokine interleukins-6 (IL-6) (Fig. 6L) and signal transducers and activators of transcription 3 (Stat3) (Fig. 6M) were observed in electrical stimulation and combination therapy groups on day 3. The IL-6 can positively influence different processes at the wound site, including stimulation of keratinocyte and fibroblast proliferation, synthesis and breakdown of ECM proteins, fibroblast chemotaxis, and regulation of the immune response (*38*), while Stats are cytoplasmic proteins that can transduce signals from a variety of growth factors and regulate target gene expression. *Stat3 *can be activated upon binding of IL-6 to its receptor and thus plays a key role in wound healing (*55*). These results further confirmed the powerful combinatorial therapeutic capabilities of the wearable patch to accelerate chronic wound healing.

## DISCUSSION

We present the development of a wireless wearable bioelectronic system consisting of a multimodal biosensor array for multiplexed monitoring of wound exudate biomarkers, a stimulus-responsive drug-loaded electroactive hydrogel, and a pair of voltage-modulated electrodes for controlled drug release and electrical stimulation. The wearable patch is fully biocompatible, mechanically flexible, stretchable, skin-conformal, and is capable of real-time selective monitoring of a panel of crucial wound biomarkers including temperature, pH, ammonium, glucose, lactate, and UA in multiple rodent models. The wearable patch demonstrated here represents a versatile platform for evaluating wound conditions and intelligent therapy and can be easily reconfigured to monitor several other metabolic and inflammatory biomarkers for various chronic wound care applications.

Despite remarkable progress in developing wearable electrochemical biosensors for continuous monitoring of circulating metabolites in interstitial fluid and human sweat (*24*, *26*, *56*–*58*), in situ wound fluid analysis remains a major clinical challenge. This is mainly due to the complex and heterogeneous composition of wound fluid (e.g., high protein levels, local and migrated cells, and exogenous factors such as bacteria) that leads to severe and unique matrix effects for most previously reported biosensors and failure in accurate measurement of the target metabolite levels in wound fluid (*31*). To mitigate such issue, here, we introduced the use of an outer porous PU-based membrane that serves as an analyte diffusion limiting layer to protect the electrode, tune response, increase long-term operational stability, linearity, and sensitivity magnitude as well as biocompatibility and mechanical stability of the sensor (*59*). Our results indicate that the wearable patch-enabled wound fluid analysis could be a promising approach to realize continuous and personalized wound metabolic monitoring in both a temporal and spatial fashion.

In addition to the multiplexed biosensors, the wearable patch is equipped with an on-demand electro-responsive drug release system, loaded with an antimicrobial and anti-inflammatory peptide. Under an applied positive voltage, the electroactive hydrogels will rapidly release the dual-function peptide that could effectively eliminate bacteria and modulate inflammatory responses in the wound site during the initial stages of healing, in a splinted excisional wound model in diabetic rats. The on-demand drug delivery can be readily modified with different electroactive hydrogels to deliver several other positively or negatively charged drugs and biomolecules (e.g., proteins, peptides, and growth factors). The integration of an electrical stimulation therapeutic module could facilitate cell motility and proliferation and ECM deposition and remodeling in the process of wound regeneration resulting in rapid and effective cutaneous wound healing.

We demonstrated promising preliminary data for multiplexed in situ metabolic monitoring. However, one limitation of the current study is the lack of a continuous wound fluid sampling and circulation system. The mixing of newly secreted analytes with the old ones delayed the sensor response, leading to a compromised temporal sensing resolution. In addition, the long-term continuous operation stability of the biosensors in wound fluid in vivo may need further improvement. Additional antifouling protective membranes could be explored to minimize the influence of complex would fluid on sensor performance during in situ use. Compared to large critical-sized wounds in large animals (e.g., pigs), the spatial biomarker mapping of wounds in rodent models did not reveal substantial spatial variations. Future investigations can focus on using a microfluidic wound fluid sampling system for efficient capture and continuous delivery of wound fluid to the sensor chamber to improve the temporal resolution of in situ biomarker detection (*27*, *56*). Moreover, to improve wearable patch’s durability, low-power electronics or energy-harvesting modules could also be implemented into the wearable platform (*60*–*63*). The clinical technology transfer of this product will require multiple further in-depth studies including preclinical biocompatibility evaluation, long-term multiplexed sensor analysis, and efficacy assessment of the closed-loop therapeutic and regenerative modules in pig models due to anatomical, physiological, and functional similarities of pig and human skin wound healing. Further in-depth studies of the cellular and molecular mechanisms behind wound regeneration upon applying the wearable patch’s combination therapy via single-cell RNA sequencing would be beneficial. Another further device development direction to benefit future evaluation is scale-up manufacturing, packaging, and reliability assessment toward first-in-human studies. We envision that the custom-engineered fully integrated wearable patch could serve as a more effective, fully controllable, and easy-to-implement platform for personalized monitoring and treatment of chronic wounds with minimal side effects.

## MATERIALS AND METHODS

### Materials and reagents

Tetrahydrofuran (THF), PVB resin BUTVAR B-98, sodium chloride, ammonium chloride, gelatin (from bovine skin), sodium thiosulfate pentahydrate, sodium bisulfite, ammonium ionophore I, aniline, CS, 1,4-butanediol diglycidyl ether (BDDE), 3,4-ethylenedioxythiophene (EDOT), poly(sodium 4-styrenesulfonate) (NaPSS), PU, glucose oxidase, uricase, chitosan, iron (III) chloride, potassium ferricyanide (III), paraformaldehyde, UA, and multiwalled carbon nanotubes (CNTs) were obtained from Sigma-Aldrich. l-lactate oxidase was purchased from Toyobo Corp. Hydrochloric acid, acetic acid, methanol, ethanol, acetone, urea, dextrose (d-glucose), and Dulbecco’s phosphate-buffered saline (DPBS) were acquired from Thermo Fisher Scientific. The SEBS polymer was obtained from Asahi Kasei Corporation. TCP-25 (GKYGFYTHVFRLKKWIQKVIDQFGE) (98% purity, acetate salt) and tetramethylrhodamine-labeled TCP-25 were purchased from CPC Scientific.

### Fabrication of the soft wearable patch

Briefly, a 300-nm-thick sacrificial layer of copper was first deposited on the silicon wafer using e-beam evaporation (CHA Industries Mark 40) at a speed of 2.5 Å s^−1^, followed by standard photolithography (Microchemicals GmbH, AZ 5214) to define the connection wires. Cr/Au/Cr (1/100/20 nm) was deposited on the sacrificial copper through e-beam evaporation of at a speed of 0.2, 0.5, and 0.2 Å/s, respectively, followed by lift-off in acetone. SEBS (200 mg ml^−1^ in toluene) was then spin-coated with a speed of 300 revolutions per minute (rpm) for 30 s. The SEBS film was cured at 70°C for 1 hour to remove toluene, and the resulting SEBS film had a thickness of ~300 μm. The copper sacrificial layer was then chemically removed by immersing the silicon wafer in copper etchant (APS-100) for 12 hours. The patch was then picked up by a polydimethylsiloxane (PDMS) stamp and rinsed with deionized (DI) water thoroughly. A thin layer of parylene (ParaTech LabTop 3000 Parylene coater) was deposited (200 nm), followed by photolithography and reactive-ion etching (RIE) (Oxford Plasmalab, 100 ICP/RIE, 30 SCCM of O_2_, 100 W, 50 mtorr, 90 s) to expose openings for sensor modifications and pin connections. Laser patterning via a 50-W CO_2_ laser cutter (Universal Laser Systems; power, 20%; speed, 50%; points per inch, 1000; and vector mode) was used to define the patch shape and outline. After sensor modification, a water-soluble tape (AQUASOL) was used to pick up the wound patch from PDMS backings for further use.

### Biosensors preparation

#### Enzymatic sensors

To increase the electrode surface area for enzymatic sensors, a nanostructured Au film was electrodeposited on Au electrodes in a solution containing 50 mM chloroauric acid and 0.1 M HCl using multipotential deposition for 1500 cycles (for each cycle, −0.9 V for 0.02 s and 0.9 V for 0.02 s). For glucose and lactate sensors, a PB layer was deposited onto the Au electrodes by 10 cycles of cyclic voltammograms (CVs) (−0.2–0.6 V versus Ag/AgCl) with a scan rate of 50 mV s^−1^ in a freshly made solution containing 2.5 mM FeCl_3_, 2.5 mM K_3_[Fe (CN)_6_], 100 mM KCl, and 100 mM HCl. For the UA sensor, a PB layer was deposited using the same approach except only one CV cycle of electrodeposition. Next, a chitosan solution was prepared by dissolving 1% chitosan in a 2% acetic acid solution followed by vigorous magnetic stirring for 1 hour. The resulting solution was then mixed with CNTs (2 mg ml^−1^) by ultrasonic agitation over 30 min to prepare a chitosan/CNT solution. To prepare all enzymatic sensors, the chitosan/CNT solution was mixed thoroughly with an enzyme solution [10 mg ml^−1^ in PBS (pH 7.2)] with a volume ratio of 2:1. Next, 1 μl of the enzyme/chitosan/CNT cocktail was drop-casted onto the PB/Au electrode and dried under 4°C. Last, the PU layer was prepared by drop-casting 4.5 μl of 15 mg ml^−1^ of PU solution in a solvent mixture containing THF and *N*,*N*′-dimethylformamide (volume ratio, 98:2) on the enzyme layer and air-dried overnight under 4°C.

#### pH sensor

The pH sensor was based on pH-sensitive polyaniline film deposited on a Au electrode. First, the working electrode was electrochemically cleaned via 10 cycles of CVs with a scan rate of 0.1 V s^−1^ in 0.5 M HCl (−0.1–0.9 V). Next, the polyaniline electro-polymerization was performed in a 50-μl solution containing 0.1 M aniline and 1 M HCl via 12 CV cycles (−0.2–1.0 V) with a scan rate of 0.1 V s^−1^. The fresh solution was then used for another 12 CV cycles. Last, pH electrodes were air-dried overnight.

#### Ammonium sensor

A PEDOT:PSS film was electrodeposited using a constant current of 0.2 mA cm^−2^ for 10 min in a solution prepared by dissolving ferrocyanide (30 mg), NaPSS (206.1 mg), and EDOT (10.7 μl) in 10 ml of DI water. Next, an NH_4_^+^ selective membrane cocktail solution was prepared by dissolving 1 mg of ammonium ionophore I, 33 mg of polyvinyl chloride, and 66 mg of bis(2-ethylhexyl)sebacate (DOS) in 660 μl of THF. A 1.5 μl of the cocktail solution was then drop-casted on the PEDOT layer to create an ammonium-selective membrane and air-dried overnight.

#### Reference electrode

To prepare the Ag/AgCl reference electrode, silver was electrodeposited at −0.2 mA for 100 s using a plating solution containing 250 mM silver nitrate, 750 mM sodium thiosulfate, and 500 mM sodium bisulfite. Ten-microliter solution of 0.1 M FeCl_3_ was dropped on the Ag electrode for 90 s. Next, a solid-state reference membrane cocktail was prepared by dissolving 78.1 mg of PVB and 50 mg of NaCl in 1 ml of methanol followed by vigorous agitation in an ultrasonic bath for 30 min. Next, a 2.5 μl of the reference cocktail membrane was drop-casted on the Ag/AgCl electrode surface and air-dried overnight.

### The characterization of multiplex biosensors

The multiplex sensor patches were characterized to evaluate their sensitivity, stability, and reproducibility in solutions of target analytes in SWF using a 1000C Multi-Potentiostat (8-channel) (CH Instruments Inc., Austin, TX, USA). The SWF solution was prepared by dissolving 584.4 mg of NaCl, 336.0 mg of NaHCO₃, 29.8 mg of KCl, 27.8 mg of CaCl_2_, and 3.30 g of bovine serum albumin in 100 ml of DI water. The enzymatic sensors were characterized chronoamperometrically in 0 to 40 mM glucose, 0 to 4 mM lactate, and 0 to 150 μM UA, at a potential of 0 V. The pH sensor calibration was performed in McIlvaine buffer solutions. Both pH and ammonium sensors were characterized electrochemically using open circuit potential.

### Drug-loaded hydrogel preparation and characterization

#### Electroactive hydrogel synthesis and 3D printing

Three hundred milligrams of CS was dissolved in 1.14 ml of 1 M NaOH under vigorous stirring. Next, 279 μl of BDDE cross-linker was added and mixed thoroughly for another 30 min. An Anton Paar MCR302 rheometer equipped with a parallel plate to perform rheological characterization. Dynamic viscosity of the samples was measured as a function of shear rate. 3D hydrogel printing was performed on the basis of a custom-designed 3D printer based on a gantry system (A3200, Aerotech) and a benchtop dispenser (Ultimus V, Nordson EFD). One hundred fifty–micrometer nozzles were used for the printing. The pump pressure was set to be 14 kPa, and the nozzle moving speed was set at 5 mm s^−1^. The printed hydrogel was placed under 60°C for 60 min to form the cross-linked network of the electroactive hydrogels. The cross-linked hydrogels were then left in DI water at 4°C for 48 hours (with water replacement every 12 hours) to obtain equilibrium swelling.

#### Drug loading and release studies

The AMP was loaded into the hydrogel by incubating swollen electroactive gel in 1.5 ml of AMP solution (2 mg ml^−1^ in DPBS) in a sealed 12-well plate under 4°C for 24 hours. Passive as well as electro-stimulated release was examined at room temperature in a DPBS solution using the wearable patch. The AMP release was quantified by measuring fluorescence signals using a Synergy HTX Multi-Mode Reader (BioTek Instruments) spectrophotometer at 570 and 583 nm.

#### Swelling studies

The initial wet weight of each prepared hydrogel was documented. The samples were then immersed in DI water, and the hydrated samples were temporarily taken out of the water and weighed at 1, 4, 8, 24, and 48 hours. The swelling ratio was calculated as the weight gain divided by the original weight before hydration.

### In vitro cell studies

#### Cell lines

Normal Adult HDF cells (Lonza) and NHEKs (Lonza) were cultured under 37°C and 5% CO_2_. Cells were passaged at 70% confluency, and a passage number of 3 to 5 was used for all studies.

#### In vitro cytocompatibility studies

The electroactive hydrogels were washed and transferred to 24-well cell culture inserts (cell culture on permeable supports). The wells were seeded with HDFs and NHEKs (1 × 10^5^ cells per well). The inserts were then placed in cell seeded 24-well plates, and cells were treated with appropriate media and incubated under 37°C and 5% CO_2_ for the course of study. A similar study was performed for wearable patches with the cells directly seeded on the patches.

#### Evaluation of cell proliferation and viability

A commercial calcein AM/ethidium homodimer-1 live/dead kit (Invitrogen) and commercial PrestoBlue assays (Thermo Fisher Scientific) were used to evaluate cell viability and cell metabolic activity, respectively. In the live/dead assay, the samples were imaged with an Axio Observer inverted microscope (ZEISS); live cells were stained green with calcein-AM, whereas dead cells were stained red with ethidium homodimer-1. Using ImageJ software, cell viability was calculated as the percentage ratio of number of live cells to the number of total cells (live + dead).

#### In vitro wound healing assay

For in vivo wound healing assay (circular wound), first, a gelatin solution was prepared by dissolving gelatin in DI water (300 mg ml^−1^) and filtered with a sterile polyethersulfone syringe filter (0.22 μm in pore size). Then, 50 μl of the solution was dropped in the center of each well in 12-well plates. Before cell seeding, the plates were kept at room temperature under sterile conditions to keep gelatin in solid condition. Next, HDF cells with a density of 1 × 10^5^ cells per well were seeded in each well and incubated at 37°C and 5% CO_2_. The inherent thermoresponsive properties of gelatin allowed slow dissolving of the gel into the media, creating a uniform-sized wound in the center of cells adhered to the plate. The medium was then replaced by fresh media after 4 hours, and the wound closure was assessed daily for up to 4 days.

### In vitro evaluation of wearable patch’s antimicrobial properties

#### Bacterial cells

Methicillin-resistant *S. aureus* [American Type Culture Collection (ATCC) BAA-2313], *P. aeruginosa* (ATCC 15442), MDR *E. coli* (ATCC BAA-2452), and *S. epidermidis* (ATCC 12228) were used for antimicrobial tests.

#### Minimum inhibitory concentration

The minimum inhibitory concentration (MIC) of TCP-25 AMP against different pathogens was evaluated by measuring bacterial optical density. First, bacteria colonies were grown on agar plates containing 15 g l^−1^ agar and 30 g l^−1^ Bacto BD tryptic soy broth (TSB) under 5% CO_2_ at 37°C for 24 hours. Next, the colonies were transferred and dispersed gently to TSB media, grown overnight in a shaker incubator at 37°C. A bacteria solution of 10^6^ CFU ml^−1^ was prepared for all antimicrobial tests. For MIC test, 200 μl of bacteria solution in TSB was cultured in 96-well plates in the presence of different AMP concentrations (0, 5, 25, 50, 100, 250, 500, and 750 μg ml^−1^) and incubated at 37°C for 24 hours. Next, the optical density of the solutions was measured, and the relative optical density (as compared to the optical density of the control sample incubated in the absence of AMP) was reported to calculate MIC.

#### CFU test

Electroactive hydrogels with and without TCP-25 AMP were placed in 24-well plates and incubated with 1 ml of bacteria solution (10^6^ CFU ml^−1^) in TSB media under 37°C and 5% CO_2_ for 18 hours. Next, each sample was removed from bacteria solution, washed gently with DPBS (3×), and then placed in microcentrifuge tubes containing 1 ml of DPBS. The tubes were vortexed vigorously at 3000 rpm for 15 min to release bacteria trapped inside the hydrogels. A series of logarithmic dilutions (10, 10^2^, 10^3^, and 10^4^) was then prepared from each solution. Twenty-microliter diluted solutions were then seeded on agar plates, followed by incubation under 37°C and 5% CO_2_ for 18 hours. The number of colonies was then recorded and reported as CFU.

#### Zone of inhibition

A 100-μl bacteria solution (10^6^ CFU ml^−1^) was dispersed uniformly each agar plate. Next, sterilized electroactive hydrogel disks (6 mm in diameter) loaded with AMP or without AMP were placed into 9-mm holes created in agar plates. The zone of inhibition was measured after 18 hours.

### Numerical electrical field simulation

Simulation of the electric field generated during electrical stimulation was conducted by using the commercial software COMSOL Multiphysics through finite element method. Tetrahedral elements allowed modeling of the electric field in 3D space with testified accuracy. The electric field is described by

$$\begin{array}{c} \nabla \cdot D=\rho \\ E=-\nabla V \end{array}$$

where *D*, ρ, *E*, and *V* denote the electric displacement field, charge density, electric field, and electric potential.

The device was fixed at the middle of a cubic computational domain. The side length of the computational domain was 100 mm. The relative permittivity above and below the device was set to be 1 and 76.8, respectively. The boundary condition for the computational domain was set by

n⋅D=0

where *n* indicates the normal to surface of the boundary. The potential of the anode was set to be 1 V, and the potential of the ground electrode was set to be 0 V.

### Wireless system integration of the wearable patch

A four-layer FPCB with a rounded rectangle (36.5 mm by 25.5 mm) geometry was designed using EAGLE CAD. The sensor patch was interfaced directly underneath the FPCB through a rectangular cutout (12 mm by 3.8 mm). The power management circuitry consists of a magnetic reed switch (MK24-B-3, Standex-Meder Electronics) and a voltage regulator (ADP162, Analog Devices). The electrical stimulation and drug delivery circuitry consist of a series voltage reference (ISL60002, Renesas Electronics), an operational amplifier square wave generator circuit (MAX40108, Maxim Integrated), and a switch array (TMUX1112, Texas Instruments). The potentiometric, amperometric, and temperature sensor interface circuitry consists of a voltage buffer array (MAX40018, Maxim Integrated), a switch array (TMUX1112, Texas Instruments), a voltage divider, and an electrochemical analog front-end (AD5941, Analog Devices). A programmable system on chip Bluetooth Low Energy (BLE) module (CYBLE-222014, Infineon Technologies) was used for data processing and wireless communication. The fully integrated wearable device was attached to the mice or rats using a 3M double sided tape and fixed with Mastisol liquid adhesive to enable strong adhesion, allowing the animals to move freely over a prolonged period.

### Characterization of adhesion of wearable patch

The wearable patch was attached to chicken skin (2 cm by 2 cm) using Mastisol liquid adhesive and 3M double sided tape as described previously. A standard T-peel test was then performed according to American Society for Testing and Materials D1876 using a mechanical tester to evaluate patch adhesion to skin. Tegaderm adhesive (3M) was used as control.

### Animal studies

#### In vivo biodegradation and biocompatibility

To assess biodegradation and biocompatibility of the wearable patch, a rat subcutaneous implantation model was used. After anesthesia and analgesia using 2.5% (v/v) isoflurane, buprenorphine (1 mg kg^−1^), ketoprofen (5 mg kg^−1^), and bupivacaine (1 mg kg^−1^), 10-mm incisions in dorsal skin were created to form subcutaneous pockets on the back of Wistar rats (200 to 250 g; Charles River Laboratories, Wilmington, MA, USA). Next, samples were implanted into each pocket according to the protocol approved by the Institutional Animal Care and Use Committee (protocol no. IA20-1800) at California Institute of Technology. Animals were then euthanized after 14 and 56 days, and the samples were explanted with their surrounding tissues for further analysis.

#### Multiplexed wound biomarker monitoring in vivo

The on-body multiplex wound biomarker monitoring was performed using a diabetic wound model in db/db mice (BKS.Cg-Dock7^m^ +/+ Lepr^db^/J mice, The Jackson Laboratory, Bar Harbor, ME, USA). After anesthesia and analgesia, a 10-mm full-thickness wounds (through to the level of the panniculus carnosus muscle) was created on the dorsum of mice using a surgical blade. A silicon 12-mm-diameter splint (Grace Bio-Labs, Bend, OR, USA) was placed on the wound area, secured with cyanoacrylate glue, and then fixed using Ethilon 5-0 sterile sutures (Nylon). The wearable patch was then placed on the wound and secured on the wound area using Tegaderm transparent film dressing (3M). The data from the wearable patch were wirelessly recorded. In the case of the infected wound, a mixture of bacteria solution (50-μl solution, 10^6^ CFU ml^−1^ MRSA, and 10^6^ CFU ml^−1^ *P. aeruginosa*) was applied into the wound area on day 4 after surgery. For the fasting experiments, the animals were fasted for 24 hours (only water was provided). One group of fasting animals were used for injection study. In this case, a 400 mM glucose solution in DBPS (based on body weight) was administered into the mouse tail vein to spark ~10 mM increase in blood glucose level. The in vivo sensor readings from the wearable patch were obtained from 30 min before injection and continued until 270 min after injection. For the fasting/feeding experiment, the animals were fasted for 24 hours, followed by feeding with protein rich laboratory rodent diet 5001 (LabDiet). For the food feeding study, the wearable patch was tested before fasting, after 24-hour fasting, and 6 hours of fasting/feeding.

#### Spatial and temporal monitoring of critical-sized wounds

Similar to multiplexed wound biomarker monitoring, critical-sized wounds (35 mm in diameter) were created in ZDF obese fa/fa diabetic rats (The Jackson Laboratory, Bar Harbor, ME, USA). Next, the sensor array patch was applied on the wound and secured by using 3M Tegaderm dressing. Simultaneous sensor readings were recorded daily for both infected and noninfected wounds before and after treatment. For the infection, a similar mixture of bacteria solution (100 μl solution, 10^6^ CFU ml^−1^ MRSA, and 10^6^ CFU ml^−1^ *P. aeruginosa*) was applied into the wound area on day 2 after surgery. During the in vivo trial, the data from the wearable patch were wirelessly recorded.

#### Evaluation of wearable patch-facilitated chronic wound healing in vivo

A 10-mm full-thickness wound was created in the ZDF obese fa/fa rat’s dorsal skin, and the wearable patch was placed on the wound. Four different rat groups were tested with different treatments offered by the wearable patch: negative control, drug release, electrical stimulation, and combination therapy. The animals were euthanized, and the tissue samples were explanted on days 4 and 14 after surgery and processed for further analysis. The adhesion of patches on animals during the course of study was monitored.

#### In vivo antimicrobial, histological, and immunohistofluorescent evaluations

For in vivo biocompatibility assessment, upon explantation, samples were fixed in 4% paraformaldehyde under 4°C overnight, washed thoroughly with DPBS (5×), and then incubated in 30% sucrose overnight (4°C). The samples were then mounted in optimal cutting temperature compound (Thermo Fisher Scientific) followed by flash freezing in liquid nitrogen (N_2_) and cryosectioning (10-μm sections). Hematoxylin and eosin and immunohistochemistry (IHC) staining were performed on cryosections. For IHC staining, two primary antibodies [anti-CD3 [SP7] (ab16669) and anti-CD68 (ab31630), Abcam] and two secondary antibodies [donkey anti-mouse, Alexa Fluor 568– and goat anti-rabbit, Alexa Fluor 488–conjugated antibodies; Invitrogen] were used. Upon antibody staining, the samples were counterstained against 4′,6-diamidino-2-phenylindole for cell nuclei visualization. The stained slides were then mounted with ProLong Diamond Antifade Mountant (Invitrogen) and imaged using an LSM 800 confocal laser scanning microscope (ZEISS).

For regeneration studies, the bacteria samples were first isolated from the wound bed and assessed via CFU assay as described earlier. The wound samples were then explanted with the adjacent tissue, processed, sectioned, and stained via MTC staining and IHC. For IHC staining, different primary antibodies including recombinant anti-cytokeratin 5 antibody [SP27] (ab64081, Abcam), anti–NF-κB p65 (phospho S276) antibody (ab194726, Abcam), human/mouse/rat PTEN Alexa Fluor 647–conjugated antibody (IC847R, R&D Systems), and cytokeratin 14 monoclonal antibody (LL002, Thermo Fisher Scientific) and similar secondary antibodies were used. Upon staining, the samples were mounted with antifade mountant and visualized with a confocal microscope.

#### qRT-PCR analysis

RNA was isolated from wound tissue samples using the RNeasy Plus Micro Kit (QIAGEN). The RNA quantity and quality were assessed using a NanoDrop 2000/2000c spectrophotometer at 260/280 nm wavelengths. Next, the complementary DNA (cDNA) was synthesized using the QuantiTect Reverse Transcription Kit (QIAGEN). Gene expression was performed using a TaqMan Universal PCR Master Mix (Thermo Fisher Scientific). TaqMan Array Plates for rat wound healing gene expression were used where a library of genes was screened. The cDNAs synthesized in the previous step were then added to each plate and followed by quantitative analysis using a QuantStudio 3 Real-Time PCR system (Applied Biosystems).

## Supplementary Materials

### This PDF file includes:

Materials and Methods Notes S1 and S2 Figs. S1 to S32 Legend for movie S1 References

## References

[1] G. C. Gurtner, S. Werner, Y. Barrandon, M. T. Longaker, Wound repair and regeneration. Nature 453, 314–321 (2008). 18480812 10.1038/nature07039

[2] S. A. Eming, P. Martin, M. Tomic-Canic, Wound repair and regeneration: Mechanisms, signaling, and translation. Sci. Transl. Med. 6, 265sr6 (2014). 25473038 10.1126/scitranslmed.3009337 PMC4973620

[3] B. K. Sun, Z. Siprashvili, P. A. Khavari, Advances in skin grafting and treatment of cutaneous wounds. Science 346, 941–945 (2014). 25414301 10.1126/science.1253836

[4] C. E. Fife, M. J. Carter, Wound care outcomes and associated cost among patients treated in US outpatient wound centers: Data from the US wound registry. Wounds 24, 10–17 (2012). 25875947

[5] D. G. Armstrong, A. J. M. Boulton, S. A. Bus, Diabetic foot ulcers and their recurrence. N. Engl. J. Med. 376, 2367–2375 (2017). 28614678 10.1056/NEJMra1615439

[6] R. G. Frykberg, J. Banks, Challenges in the treatment of chronic wounds. Adv. Wound Care 4, 560–582 (2015). 10.1089/wound.2015.0635 PMC4528992 26339534

[7] E. Eriksson, P. Y. Liu, G. S. Schultz, M. M. Martins-Green, R. Tanaka, D. Weir, L. J. Gould, D. G. Armstrong, G. W. Gibbons, R. Wolcott, O. O. Olutoye, R. S. Kirsner, G. C. Gurtner, Chronic wounds: Treatment consensus. Wound Repair Regen. 30, 156–171 (2022). 35130362 10.1111/wrr.12994 PMC9305950

[8] J. M. A. Blair, M. A. Webber, A. J. Baylay, D. O. Ogbolu, L. J. V. Piddock, Molecular mechanisms of antibiotic resistance. Nat. Rev. Microbiol. 13, 42–51 (2015). 25435309 10.1038/nrmicro3380

[9] G. Thakral, J. LaFontaine, B. Najafi, T. K. Talal, P. Kim, L. A. Lavery, Electrical stimulation to accelerate wound healing. Diabet. Foot Ankle 4, 22081 (2013). 10.3402/dfa.v4i0.22081 PMC3776323 24049559

[10] L. C. Kloth, Electrical stimulation technologies for wound healing. Adv. Wound Care 3, 81–90 (2014). 10.1089/wound.2013.0459 PMC3929255 24761348

[11] S. Patel, A. Maheshwari, A. Chandra, Biomarkers for wound healing and their evaluation. J. Wound Care 25, 46–55 (2016). 26762498 10.12968/jowc.2016.25.1.46

[12] L. E. Lindley, O. Stojadinovic, I. Pastar, M. Tomic-Canic, Biology and biomarkers for wound healing. Plast. Reconstr. Surg. 138, 18S–28S (2016). 27556760 10.1097/PRS.0000000000002682 PMC4998971

[13] K. F. Cutting, Wound exudate: Composition and functions. Br. J. Community Nurs. 8, S4–S9 (2003). 14685963 10.12968/bjcn.2003.8.sup3.11577

[14] R. A. Nery, B. S. Kahlow, T. L. Skare, F. I. Tabushi, A. d. A. e. Castro, Uric acid and tissue repair. Arq. Bras. Cir. Dig. 28, 290–292 (2015). 26734804 10.1590/S0102-6720201500040018 PMC4755186

[15] S. Britland, O. Ross-Smith, H. Jamil, A. G. Smith, K. Vowden, P. Vowden, The lactate conundrum in wound healing: Clinical and experimental findings indicate the requirement for a rapid point-of-care diagnostic. Biotechnol. Prog. 28, 917–924 (2012). 22581665 10.1002/btpr.1561

[16] T. Hirsch, M. Spielmann, B. Zuhaili, T. Koehler, M. Fossum, H.-U. Steinau, F. Yao, L. Steinstraesser, A. B. Onderdonk, E. Eriksson, Enhanced susceptibility to infections in a diabetic wound healing model. BMC Surg. 8, 5 (2008). 18312623 10.1186/1471-2482-8-5 PMC2276479

[17] C. Xu, Y. Yang, W. Gao, Skin-interfaced sensors in digital medicine: From materials to applications. Matter 2, 1414–1445 (2020). 32510052 10.1016/j.matt.2020.03.020 PMC7274218

[18] T. R. Ray, J. Choi, A. J. Bandodkar, S. Krishnan, P. Gutruf, L. Tian, R. Ghaffari, J. A. Rogers, Bio-integrated wearable systems: A comprehensive review. Chem. Rev. 119, 5461–5533 (2019). 30689360 10.1021/acs.chemrev.8b00573

[19] J. Kim, A. S. Campbell, B. E.-F. de Ávila, J. Wang, Wearable biosensors for healthcare monitoring. Nat. Biotechnol. 37, 389–406 (2019). 30804534 10.1038/s41587-019-0045-y PMC8183422

[20] Y. Yang, W. Gao, Wearable and flexible electronics for continuous molecular monitoring. Chem. Soc. Rev. 48, 1465–1491 (2019). 29611861 10.1039/c7cs00730b

[21] T. Someya, Z. Bao, G. G. Malliaras, The rise of plastic bioelectronics. Nature 540, 379–385 (2016). 27974769 10.1038/nature21004

[22] Z. Zhou, K. Chen, X. Li, S. Zhang, Y. Wu, Y. Zhou, K. Meng, C. Sun, Q. He, W. Fan, E. Fan, Z. Lin, X. Tan, W. Deng, J. Yang, J. Chen, Sign-to-speech translation using machine-learning-assisted stretchable sensor arrays. Nat. Electron. 3, 571–578 (2020).

[23] Y. Zhou, X. Zhao, J. Xu, Y. Fang, G. Chen, Y. Song, S. Li, J. Chen, Giant magnetoelastic effect in soft systems for bioelectronics. Nat. Mater. 20, 1670–1676 (2021). 34594013 10.1038/s41563-021-01093-1

[24] W. Gao, S. Emaminejad, H. Y. Y. Nyein, S. Challa, K. Chen, A. Peck, H. M. Fahad, H. Ota, H. Shiraki, D. Kiriya, D.-H. Lien, G. A. Brooks, R. W. Davis, A. Javey, Fully integrated wearable sensor arrays for multiplexed in situ perspiration analysis. Nature 529, 509–514 (2016). 26819044 10.1038/nature16521 PMC4996079

[25] T. R. Ray, M. Ivanovic, P. M. Curtis, D. Franklin, K. Guventurk, W. J. Jeang, J. Chafetz, H. Gaertner, G. Young, S. Rebollo, J. B. Model, S. P. Lee, J. Ciraldo, J. T. Reeder, A. Hourlier-Fargette, A. J. Bandodkar, J. Choi, A. J. Aranyosi, R. Ghaffari, S. A. McColley, S. Haymond, J. A. Rogers, Soft, skin-interfaced sweat stickers for cystic fibrosis diagnosis and management. Sci. Transl. Med. 13, eabd8109 (2021). 33790027 10.1126/scitranslmed.abd8109 PMC8351625

[26] H. Lee, T. K. Choi, Y. B. Lee, H. R. Cho, R. Ghaffari, L. Wang, H. J. Choi, T. D. Chung, N. Lu, T. Hyeon, S. H. Choi, D.-H. Kim, A graphene-based electrochemical device with thermoresponsive microneedles for diabetes monitoring and therapy. Nat. Nanotechnol. 11, 566–572 (2016). 26999482 10.1038/nnano.2016.38

[27] A. Koh, D. Kang, Y. Xue, S. Lee, R. M. Pielak, J. Kim, T. Hwang, S. Min, A. Banks, P. Bastien, M. C. Manco, L. Wang, K. R. Ammann, K.-I. Jang, P. Won, S. Han, R. Ghaffari, U. Paik, M. J. Slepian, G. Balooch, Y. Huang, J. A. Rogers, A soft, wearable microfluidic device for the capture, storage, and colorimetric sensing of sweat. Sci. Transl. Med. 8, 366ra165 (2016). 10.1126/scitranslmed.aaf2593 PMC5429097 27881826

[28] F. Tehrani, H. Teymourian, B. Wuerstle, J. Kavner, R. Patel, A. Furmidge, R. Aghavali, H. Hosseini-Toudeshki, C. Brown, F. Zhang, K. Mahato, Z. Li, A. Barfidokht, L. Yin, P. Warren, N. Huang, Z. Patel, P. P. Mercier, J. Wang, An integrated wearable microneedle array for the continuous monitoring of multiple biomarkers in interstitial fluid. Nat. Biomed. Eng. 6, 1214–1224 (2022). 35534575 10.1038/s41551-022-00887-1

[29] J. R. Sempionatto, M. Lin, L. Yin, E. De la paz, K. Pei, T. Sonsa-ard, A. N. de Loyola Silva, A. A. Khorshed, F. Zhang, N. Tostado, S. Xu, J. Wang, An epidermal patch for the simultaneous monitoring of haemodynamic and metabolic biomarkers. Nat. Biomed. Eng. 5, 737–748 (2021). 33589782 10.1038/s41551-021-00685-1

[30] C. Wang, X. Li, H. Hu, L. Zhang, Z. Huang, M. Lin, Z. Zhang, Z. Yin, B. Huang, H. Gong, S. Bhaskaran, Y. Gu, M. Makihata, Y. Guo, Y. Lei, Y. Chen, C. Wang, Y. Li, T. Zhang, Z. Chen, A. P. Pisano, L. Zhang, Q. Zhou, S. Xu, Monitoring of the central blood pressure waveform via a conformal ultrasonic device. Nat. Biomed. Eng. 2, 687–695 (2018). 30906648 10.1038/s41551-018-0287-x PMC6428206

[31] C. Wang, E. Shirzaei Sani, W. Gao, Wearable bioelectronics for chronic wound management. Adv. Funct. Mater. 32, 2111022 (2022). 36186921 10.1002/adfm.202111022 PMC9518812

[32] A. A. Trotsyuk, S. Niu, D. Henn, K. Chen, C.-C. Shih, M. R. Larson, A. M. Mermin-Bunnell, S. Mittal, J.-C. Lai, A. Saberi, E. Beard, S. Jing, D. Zhong, S. R. Steele, K. Sun, T. Jain, E. Zhao, C. R. Neimeth, W. G. Viana, J. Tang, D.Sivaraj, J. Padmanabhan, M. Rodrigues, D. P. Perrault, A. Chattopadhyay, Z. N. Maan, M. C. Leeolou, C. A. Bonham, S. H. Kwon, H. C. Kussie, K. S. Fischer, G. Gurusankar, K. Liang, K. Zhang, R. Nag, M. P. Snyder, M. Januszyk, G. C. Gurtner, Z. Bao, Wireless closed-loop smart bandage for chronic wound management and accelerated tissue regeneration. bioRxiv 2022.01.16.476432 (2022).

[33] R. Dong, B. Guo, Smart wound dressings for wound healing. Nano Today 41, 101290 (2021).

[34] Z. Xiong, S. Achavananthadith, S. Lian, L. E. Madden, Z. X. Ong, W. Chua, V. Kalidasan, Z. Li, Z. Liu, P. Singh, H. Yang, S. P. Heussler, S. M. P. Kalaiselvi, M. B. H. Breese, H. Yao, Y. Gao, K. Sanmugam, B. C. K. Tee, P.-Y. Chen, W. Loke, C. T. Lim, G. S. H. Chiang, B. Y. Tan, H. Li, D. L. Becker, J. S. Ho, A wireless and battery-free wound infection sensor based on DNA hydrogel. Sci. Adv. 7, eabj1617 (2021). 34797719 10.1126/sciadv.abj1617 PMC8604401

[35] V. Kalidasan, X. Yang, Z. Xiong, R. R. Li, H. Yao, H. Godaba, S. Obuobi, P. Singh, X. Guan, X. Tian, S. A. Kurt, Z. Li, D. Mukherjee, R. Rajarethinam, C. S. Chong, J.-W. Wang, P. L. R. Ee, W. Loke, B. C. K. Tee, J. Ouyang, C. J. Charles, J. S. Ho, Wirelessly operated bioelectronic sutures for the monitoring of deep surgical wounds. Nat. Biomed. Eng. 5, 1217–1227 (2021). 34654900 10.1038/s41551-021-00802-0

[36] Y. Gao, D. T. Nguyen, T. Yeo, S. B. Lim, W. X. Tan, L. E. Madden, L. Jin, J. Y. K. Long, F. A. B. Aloweni, Y. J. A. Liew, M. L. L. Tan, S. Y. Ang, S. D. Maniya, I. Abdelwahab, K. P. Loh, C.-H. Chen, D. L. Becker, D. Leavesley, J. S. Ho, C. T. Lim, A flexible multiplexed immunosensor for point-of-care in situ wound monitoring. Sci. Adv. 7, eabg9614 (2021). 34020961 10.1126/sciadv.abg9614 PMC8139589

[37] P. Kassal, J. Kim, R. Kumar, W. R. de Araujo, I. M. Steinberg, M. D. Steinberg, J. Wang, Smart bandage with wireless connectivity for uric acid biosensing as an indicator of wound status. Electrochem. Commun. 56, 6–10 (2015).

[38] M. Puthia, M. Butrym, J. Petrlova, A.-C. Strömdahl, M. Å. Andersson, S. Kjellström, A. Schmidtchen, A dual-action peptide-containing hydrogel targets wound infection and inflammation. Sci. Transl. Med. 12, eaax6601 (2020). 31894104 10.1126/scitranslmed.aax6601

[39] T. Tanaka, I. Nishio, S.-T. Sun, S. Ueno-Nishio, Collapse of gels in an electric field. Science 218, 467–469 (1982). 17808541 10.1126/science.218.4571.467

[40] C. M. Proctor, A. Slézia, A. Kaszas, A. Ghestem, I. del Agua, A.-M. Pappa, C. Bernard, A. Williamson, G. G. Malliaras, Electrophoretic drug delivery for seizure control. Sci. Adv. 4, eaau1291 (2018). 30167463 10.1126/sciadv.aau1291 PMC6114990

[41] B. Song, Y. Gu, J. Pu, B. Reid, Z. Zhao, M. Zhao, Application of direct current electric fields to cells and tissues in vitro and modulation of wound electric field in vivo. Nat. Protoc. 2, 1479–1489 (2007). 17545984 10.1038/nprot.2007.205

[42] M. Zhao, B. Song, J. Pu, T. Wada, B. Reid, G. Tai, F. Wang, A. Guo, P. Walczysko, Y. Gu, T. Sasaki, A. Suzuki, J. V. Forrester, H. R. Bourne, P. N. Devreotes, C. D. McCaig, J. M. Penninger, Electrical signals control wound healing through phosphatidylinositol-3-OH kinase-γ and PTEN. Nature 442, 457–460 (2006). 16871217 10.1038/nature04925

[43] D. J. Cohen, W. James Nelson, M. M. Maharbiz, Galvanotactic control of collective cell migration in epithelial monolayers. Nat. Mater. 13, 409–417 (2014). 24608142 10.1038/nmat3891

[44] A. Chanmugam, D. Langemo, K. Thomason, J. Haan, E. A. Altenburger, A. Tippett, L. Henderson, T. A. Zortman, Relative temperature maximum in wound infection and inflammation as compared with a control subject using long-wave infrared thermography. Adv. Skin Wound Care 30, 406–414 (2017). 28817451 10.1097/01.ASW.0000522161.13573.62

[45] M. L. Fernandez, Z. Upton, H. Edwards, K. Finlayson, G. K. Shooter, Elevated uric acid correlates with wound severity. Int. Wound J. 9, 139–149 (2012). 21973196 10.1111/j.1742-481X.2011.00870.x PMC7951012

[46] G. Tegl, D. Schiffer, E. Sigl, A. Heinzle, G. M. Guebitz, Biomarkers for infection: Enzymes, microbes, and metabolites. Appl. Microbiol. Biotechnol. 99, 4595–4614 (2015). 25952112 10.1007/s00253-015-6637-7

[47] M. Digirolamo, F. D. Newby, J. Lovejoy, Lactate production in adipose tissue; a regulated function with extra-adipose implications. FASEB J. 6, 2405–2412 (1992). 1563593 10.1096/fasebj.6.7.1563593

[48] I. Sakakibara, T. Fujino, M. Ishii, T. Tanaka, T. Shimosawa, S. Miura, W. Zhang, Y. Tokutake, J. Yamamoto, M. Awano, S. Iwasaki, T. Motoike, M. Okamura, T. Inagaki, K. Kita, O. Ezaki, M. Naito, T. Kuwaki, S. Chohnan, T. T. Yamamoto, R. E. Hammer, T. Kodama, M. Yanagisawa, J. Sakai, Fasting-induced hypothermia and reduced energy production in mice lacking acetyl-CoA synthetase 2. Cell Metab. 9, 191–202 (2009). 19187775 10.1016/j.cmet.2008.12.008

[49] D. R. Griffin, M. M. Archang, C.-H. Kuan, W. M. Weaver, J. S. Weinstein, A. C. Feng, A. Ruccia, E. Sideris, V. Ragkousis, J. Koh, M. V. Plikus, D. Di Carlo, T. Segura, P. O. Scumpia, Activating an adaptive immune response from a hydrogel scaffold imparts regenerative wound healing. Nat. Mater. 20, 560–569 (2021). 33168979 10.1038/s41563-020-00844-w PMC8005402

[50] Y. R. Park, M. T. Sultan, H. J. Park, J. M. Lee, H. W. Ju, O. J. Lee, D. J. Lee, D. L. Kaplan, C. H. Park, NF-κB signaling is key in the wound healing processes of silk fibroin. Acta Biomater. 67, 183–195 (2018). 29242162 10.1016/j.actbio.2017.12.006

[51] A. Leask, Potential therapeutic targets for cardiac Fibrosis. Circ. Res. 106, 1675–1680 (2010). 20538689 10.1161/CIRCRESAHA.110.217737

[52] C. Profyris, C. Tziotzios, I. Do Vale, Cutaneous scarring: Pathophysiology, molecular mechanisms, and scar reduction therapeutics Part I. The molecular basis of scar formation. J. Am. Acad. Dermatol. 66, 1–10; quiz 11–2 (2012). 22177631 10.1016/j.jaad.2011.05.055

[53] I. Gerling, C. Nejman, N. K. Chatterjee, Effect of coxsackievirus B4 infection in mice on expression of 64,000-Mr autoantigen and glucose sensitivity of islets before development of hyperglycemia. Diabetes 37, 1419–1425 (1988). 2843410 10.2337/diab.37.10.1419

[54] T. M. Simone, C. E. Higgins, R.-P. Czekay, B. K. Law, S. P. Higgins, J. Archambeault, S. M. Kutz, P. J. Higgins, SERPINE1: A molecular switch in the proliferation-migration dichotomy in wound-"activated" keratinocytes. Adv. Wound Care 3, 281–290 (2014). 10.1089/wound.2013.0512 PMC3955966 24669362

[55] H. Yu, R. Jove, The STATs of cancer--new molecular targets come of age. Nat. Rev. Cancer 4, 97–105 (2004). 14964307 10.1038/nrc1275

[56] M. Wang, Y. Yang, J. Min, Y. Song, J. Tu, D. Mukasa, C. Ye, C. Xu, N. Heflin, J. S. McCune, T. K. Hsiai, Z. Li, W. Gao, A wearable electrochemical biosensor for the monitoring of metabolites and nutrients. Nat. Biomed. Eng. 6, 1225–1235 (2022). 35970928 10.1038/s41551-022-00916-z PMC10432133

[57] Y. Yang, Y. Song, X. Bo, J. Min, O. S. Pak, L. Zhu, M. Wang, J. Tu, A. Kogan, H. Zhang, T. K. Hsiai, Z. Li, W. Gao, A laser-engraved wearable sensor for sensitive detection of uric acid and tyrosine in sweat. Nat. Biotechnol. 38, 217–224 (2020). 31768044 10.1038/s41587-019-0321-x

[58] A. J. Bandodkar, P. Gutruf, J. Choi, K. Lee, Y. Sekine, J. T. Reeder, W. J. Jeang, A. J. Aranyosi, S. P. Lee, J. B. Model, R. Ghaffari, C. J. Su, J. P. Leshock, T. Ray, A. Verrillo, K. Thomas, V. Krishnamurthi, S. Han, J. Kim, S. Krishnan, T. Hang, J. A. Rogers, Battery-free, skin-interfaced microfluidic/electronic systems for simultaneous electrochemical, colorimetric, and volumetric analysis of sweat. Sci. Adv. 5, eaav3294 (2019). 30746477 10.1126/sciadv.aav3294 PMC6357758

[59] C. A. P. Quinn, R. E. Connor, A. Heller, Biocompatible, glucose-permeable hydrogel for in situ coating of implantable biosensors. Biomaterials 18, 1665–1670 (1997). 9613815 10.1016/s0142-9612(97)00125-7

[60] Y. Yu, J. Nassar, C. Xu, J. Min, Y. Yang, A. Dai, R. Doshi, A. Huang, Y. Song, R. Gehlhar, A. D. Ames, W. Gao, Biofuel-powered soft electronic skin with multiplexed and wireless sensing for human-machine interfaces. Sci. Robot. 5, eaaz7946 (2020). 32607455 10.1126/scirobotics.aaz7946 PMC7326328

[61] Y. Song, J. Min, Y. Yu, H. Wang, Y. Yang, H. Zhang, W. Gao, Wireless battery-free wearable sweat sensor powered by human motion. Sci. Adv. 6, eaay9842 (2020). 32998888 10.1126/sciadv.aay9842 PMC7527225

[62] S. Park, S. W. Heo, W. Lee, D. Inoue, Z. Jiang, K. Yu, H. Jinno, D. Hashizume, M. Sekino, T. Yokota, K. Fukuda, K. Tajima, T. Someya, Self-powered ultra-flexible electronics via nano-grating-patterned organic photovoltaics. Nature 561, 516–521 (2018). 30258137 10.1038/s41586-018-0536-x

[63] J. Chen, Y. Huang, N. Zhang, H. Zou, R. Liu, C. Tao, X. Fan, Z. L. Wang, Micro-cable structured textile for simultaneously harvesting solar and mechanical energy. Nat. Energy 1, 16138 (2016).

[64] S. S. Veidal, E. Vassiliadis, N. Barascuk, C. Zhang, T. Segovia-Silvestre, L. Klickstein, M. R. Larsen, P. Qvist, C. Christiansen, B. Vainer, M. A. Karsdal, Matrix metalloproteinase-9-mediated type III collagen degradation as a novel serological biochemical marker for liver fibrogenesis. Liver Int. 30, 1293–1304 (2010). 20666994 10.1111/j.1478-3231.2010.02309.x

[65] K. Kessenbrock, V. Plaks, Z. Werb, Matrix metalloproteinases: Regulators of the tumor microenvironment. Cell 141, 52–67 (2010). 20371345 10.1016/j.cell.2010.03.015 PMC2862057

[66] Z. I. Elbialy, D. H. Assar, A. Abdelnaby, S. A. Asa, E. Y. Abdelhiee, S. S. Ibrahim, M. M. Abdel-Daim, R. Almeer, A. Atiba, Healing potential of Spirulina platensis for skin wounds by modulating bFGF, VEGF, TGF-ß1 and α-SMA genes expression targeting angiogenesis and scar tissue formation in the rat model. Biomed. Pharmacother. 137, 111349 (2021). 33567349 10.1016/j.biopha.2021.111349

[67] M. Presta, G. Andrés, D. Leali, P. Dell’Era, R. Ronca, Inflammatory cells and chemokines sustain FGF2-induced angiogenesis. Eur. Cytokine Netw. 20, 39–50 (2009). 19541589 10.1684/ecn.2009.0155

[68] D. Wang, J. Sai, A. Richmond, Cell surface heparan sulfate participates in CXCL1-induced signaling. Biochemistry 42, 1071–1077 (2003). 12549928 10.1021/bi026425a PMC2667446

[69] P. Bao, A. Kodra, M. Tomic-Canic, M. S. Golinko, H. P. Ehrlich, H. Brem, The role of vascular endothelial growth factor in wound healing. J. Surg. Res. 153, 347–358 (2009). 19027922 10.1016/j.jss.2008.04.023 PMC2728016

