# Supplementary material for: A stretchable wireless wearable bioelectronic system for multiplexed monitoring and combination treatment of infected chronic wounds
Source: Sci Adv. 2023 Mar 24;9(12):eadf7388. doi: 10.1126/sciadv.adf7388 (PMC10038347; doi:10.1126/sciadv.adf7388)
Supplement: Supplementary file 1 — Materials and Methods Notes S1 and S2 Figs. S1 to S32 Legend for movie S1 References [file sciadv.adf7388_sm.pdf]

Science Advances
AAAS

Supplementary Materials for

A stretchable wireless wearable bioelectronic system for multiplexed monitoring and combination treatment of infected chronic wounds

Ehsan Shirzaei Sani *et al.*

Corresponding author: Wei Gao, weigao@caltech.edu

Sci. Adv. **9**, eadf7388 (2023)
DOI: 10.1126/sciadv.adf7388

The PDF file includes:

Materials and Methods
Notes S1 and S2
Figs. S1 to S32
Legend for movie S1
References

Other Supplementary Material for this manuscript includes the following:

Movie S1

Note S1. Optimization of enzymatic sensors for wound fluid analysis

The past decades have witnessed tremendous success in developing enzymatic sensors (e.g., continuous glucose monitors) for continuous monitoring of circulating metabolites in blood, interstitial fluid (ISF), or non-invasively accessible alternative fluids (e.g., sweat, saliva, and tears). However, complex and heterogeneous composition of wound fluid (e.g. high protein levels, local and migrated cells, and exogenous factors such as bacteria) leads to severe and unique matrix effects for previously reported enzymatic sensors and failure in accurate measurement of the target metabolite levels in untreated wound fluid. There are few reports on biosensors that are able to perform continuous long-term wound fluid metabolic analysis (*31*).

Taking glucose sensors as the exemplar, we examined a number of glucose sensor configurations as demonstrated in **figs. S8** and **S9**. Instable or non-responsive glucose sensor responses were commonly observed in simulated wound fluid (SWF) samples or wound fluid (WF) samples collected from diabetic rats. This indicates that previously reported glucose sensors suffer from severe matrix effects and fail to accurately measure the target metabolite levels in untreated WF. Moreover, high levels of metabolites in diabetic wound fluid, particularly glucose (up to 50 mM), pose another major challenge to obtain linear sensor response in the physiological concentration ranges.

Specifically, various glucose sensors with Prussian blue (PB) as the redox mediator were prepared based on the reported methods. Glucose sensors were first prepared by modifying glucose oxidase (GOx), GOx/bovine serum albumin (BSA), GOx/polyaniline (PANI), and GOx/chitosan (CS) on the top of the Au/PB electrodes. The first three types showed a poor linear relationship between amperometric responses and the physiological diabetic glucose concentration ranges (up to 50 mM) in SWF. In contrast, the GOx/CS/MWCNTs-based sensor showed good linear response, potentially due to the limited glucose diffusion in the CS matrix. All these sensors did not respond to the addition of glucose in WF, indicating the severe matrix influences of complex wound fluid to enzymatic sensor performance.

In order to increase sensor range and minimize biofouling effects, we explored the use of an outer porous membrane that serves as a diffusion limiting layer to protect the enzyme, tune response, increase operational stability, as well as enhance the linearity and sensitivity magnitude of the sensor. We next fabricated our enzymatic GOx/CS/MWCNTs glucose sensor with additional porous membrane coatings including CS, poly(ethylene glycol) diglycidyl ether (PEGDGE), Nafion, and polyurethane (PU) (**fig. S9**). As expected, the addition of diffusion layers indeed improves the sensor's linear range in SWF. However, CS, PEGDGE, and Nafion coated sensors did not show good response in wound fluid upon the addition of glucose. We observed that PU-based sensor showed the highest linearity over the wide physiological concentration range as well as high reproducibility in complex wound fluid matrix (**fig. S10**).

The optimized enzymatic sensors with PU coating possess several critical design features to operate in the wound bed environment for a prolonged period. First, enzymes such as glucose oxidase are specific for the target analyte in the wound fluid, which minimize the interference of the other biochemicals including electrolytes and metabolites. Second, the CS/CNT layer allows efficient electron transfer but reduces electrode poisoning due to interferences from undesired exogenous biochemicals in the wound fluid. Third, the PU layer improves long-term stability of sensor for accurate and stable *in vivo* function. In addition, polyurethane's biocompatibility improves *in vivo* durability of the sensor and eliminates safety concerns. Moreover, the PU mass

transport limiting membrane has excellent mechanical strength which improves the sensor physical stability.

Note S2. Analysis of gene expression in the wound healing process

During wound healing, the extracellular matrix (ECM) at the wound site undergoes dramatic reorganization. An elevated expression of collagen type I alpha 1 (Col1a1) and collagen type III alpha 1 (Col3a1) was observed in the electrical stimulation (ES) and combination therapy groups as compared to control group on day 3 (**fig. S31A** and **B)**. This can be mainly attributed to fibroblasts proliferation. In this process, substantial quantities of matrix proteins (predominantly collagen types I and III) are synthesized and deposited, resulting in improved tensile strength of the regenerated wound skin. Interestingly, we also observed a substantial increase in Col3a1 expression in the control group as compared to other groups on day 14. This is potentially due to downregulation of matrix metalloproteinase-9 (MMP-9) that resulted in ECM accumulation during the wound healing process and yielded to keloid or hypertrophic scarring in the control group (*64*). MMPs are calcium-dependent zinc-containing endopeptidases that collectively degrade and resorb all major components of the ECM (*65*). The upregulation of MMPs during the wound repair process contributed to scarless healing in the combination therapy group after 14 days (**fig. S31C)**.

Higher expression levels of fibroblast growth factor 10 (Fgf10), C-X-C motif chemokine ligand 1 (Cxcl1), and vascular endothelial growth factor A (Vegfa) were observed for the combination therapy group when compared to other groups (**fig. S31D** and **F)**. Such higher expression is associated with early angiogenesis and neovasculature formation during chronic wound healing (*66*). Angiogenesis is the process of newly formed blood vessels and plays a crucial role in supplying necessary nutrients to the new granulation tissue. Angiogenesis and neovascularization in the new ECM are triggered by several growth factors including Fgf2 and Fgf10 (*66*). Fgfs generally regulate angiogenesis *via* the recruitment of inflammatory cells that results in up-regulation of various chemokines such as chemokine (C-C motif) ligand 2 (Ccl2) and Cxcl1 (*67*). During inflammation, Cxcl chemokines regulate the timely recruitment of specific populations of leukocytes to the damage site. They are also important in angiogenesis, tumor formation, and tumor metastasis (*68*). Vegfa is also critical for enhancing angiogenesis in the early stages of wound healing, particularly by promoting endothelial cells migration (*69*). A significant expression of Mitogen-activated protein kinase 1/2 (Mapk1/2) was also observed in the combination therapy group as compared to other groups (**fig. S31G)**. Generally, Vegfa plays a key role in multiple endothelial cell-specific functions including activation of the downstream Mapk1/2 pathway, therefore promoting cell migration, proliferation, and angiogenesis.

The higher expression of Pten gene also confirms the immunohistochemistry results that the positive effect of electrical stimulation on preferential activation of voltage-gated channels facilitated cell migration and orientation (electrotaxis) (**Fig. 6E** and **fig. S31E,H)** (*42*).

Collectively, these findings confirmed that, through combination therapy, the wearable patch could modulate cell proliferation, migration, and ECM deposition and remodeling, enabling an accelerated scarless cutaneous wound healing.

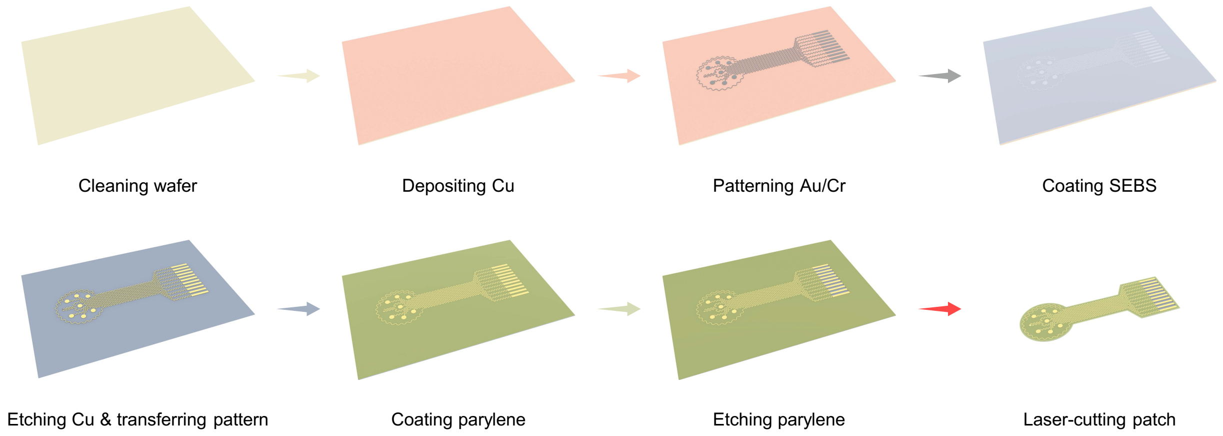

Fig. S1. The fabrication process of the stretchable wearable patch.

A B

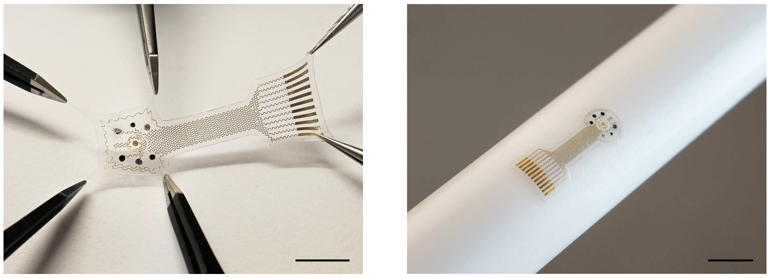

Fig. S2. Optical images of the stretchable wearable patch. (**A** and **B**) Photograph of stretchable and flexible wearable patches. Scale bars, 1 cm.

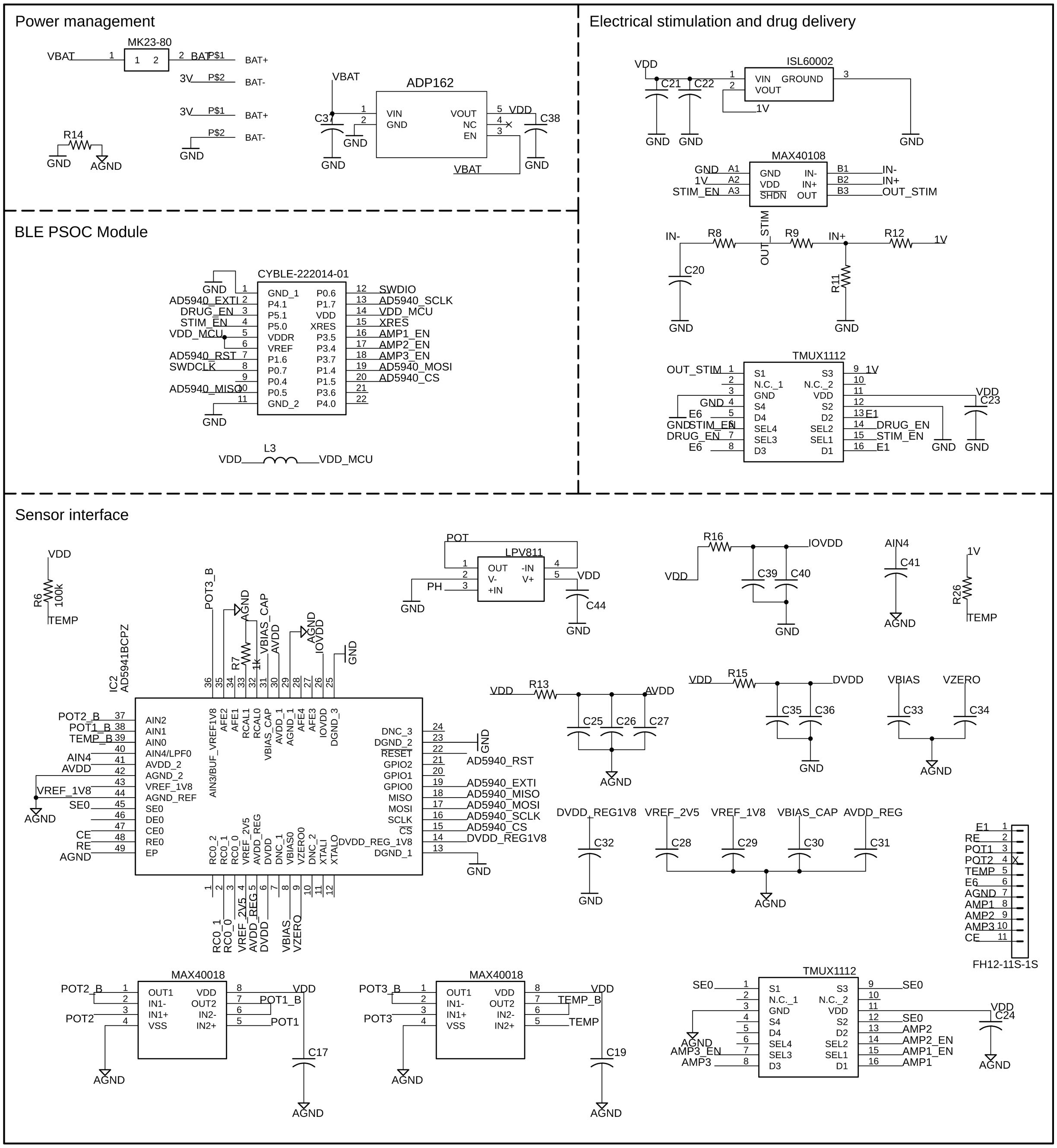

Fig. S3. Circuit diagram of the fully-integrated wearable bioelectronic system.

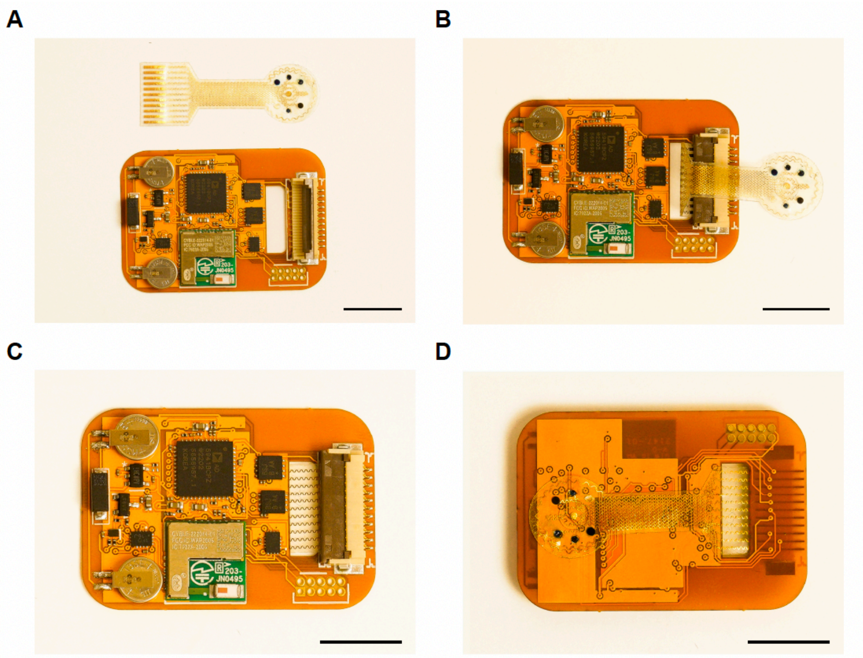

Fig. S4. Images of the assembly of the wireless wearable bioelectronic system with integrated flexible patch and a flexible printed circuit board. Scale bars, 1 cm.

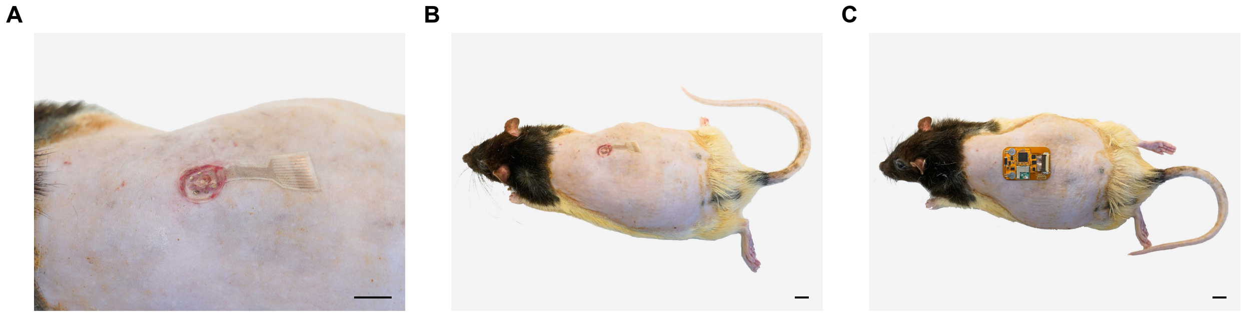

Fig. S5. Images of a wearable patch assembled on the wound of a diabetic rat. Scale bars, 1 cm.

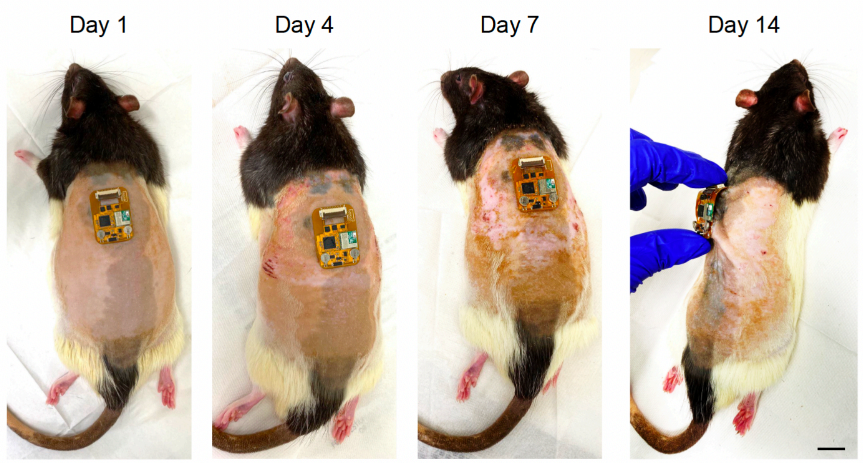

Fig. S6. Photos of a fully-integrated wearable patch on a diabetic rat with an open wound for 14 days. Scale bar, 2 cm.

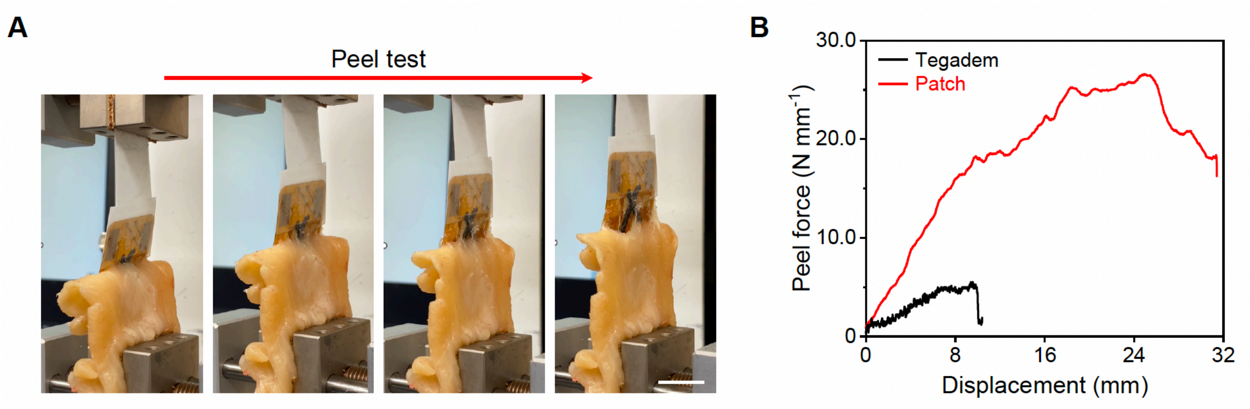

Fig. S7. Characterization of patch adhesion to chicken skin using a standard T-peel test according to American Society for Testing and Materials (ASTM) D1876. (**A**) Time lapse images of the peel test. (**B**) Tested peel force under different displacement. Scale bar, 2 cm.

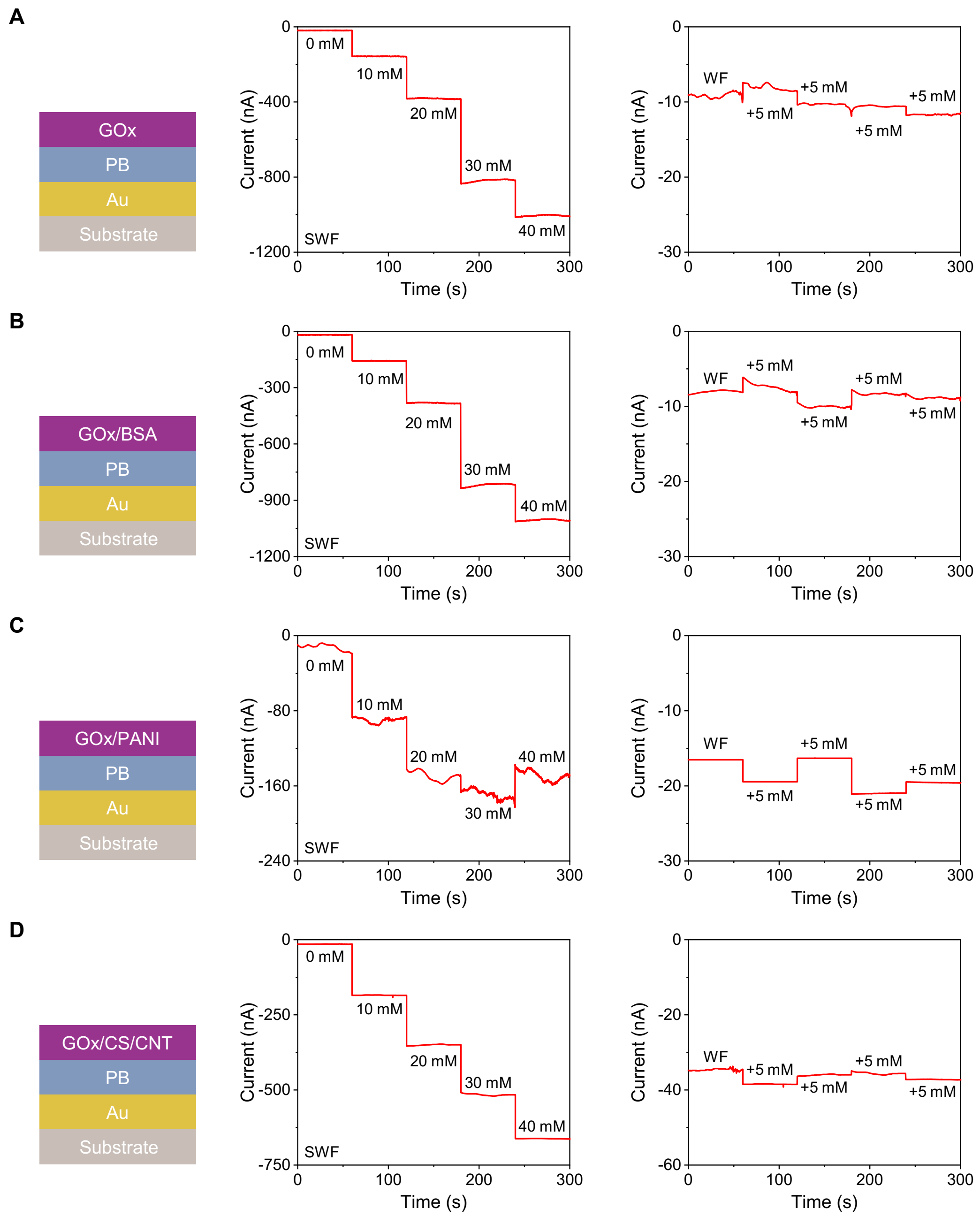

Fig. S8. The performance of enzymatic glucose sensors with different configurations in simulated wound fluid (SWF) samples and wound fluid (WF) samples collected from mice. (**A** to **D**) Schematic and performance of the glucose sensors based on glucose oxidase (GOx) (**A**), GOx/bovine serum albumin (BSA) (**B**), GOx/polyaniline (PANI) (**C**), and GOx/chitosan (CS)/multiwalled carbon nanotubes (MWCNTs) (**D**) in SWF and WF. Prussian blue (PB) was used as the redox mediator in all cases. The WF samples were collected after 24 hours of fasting.

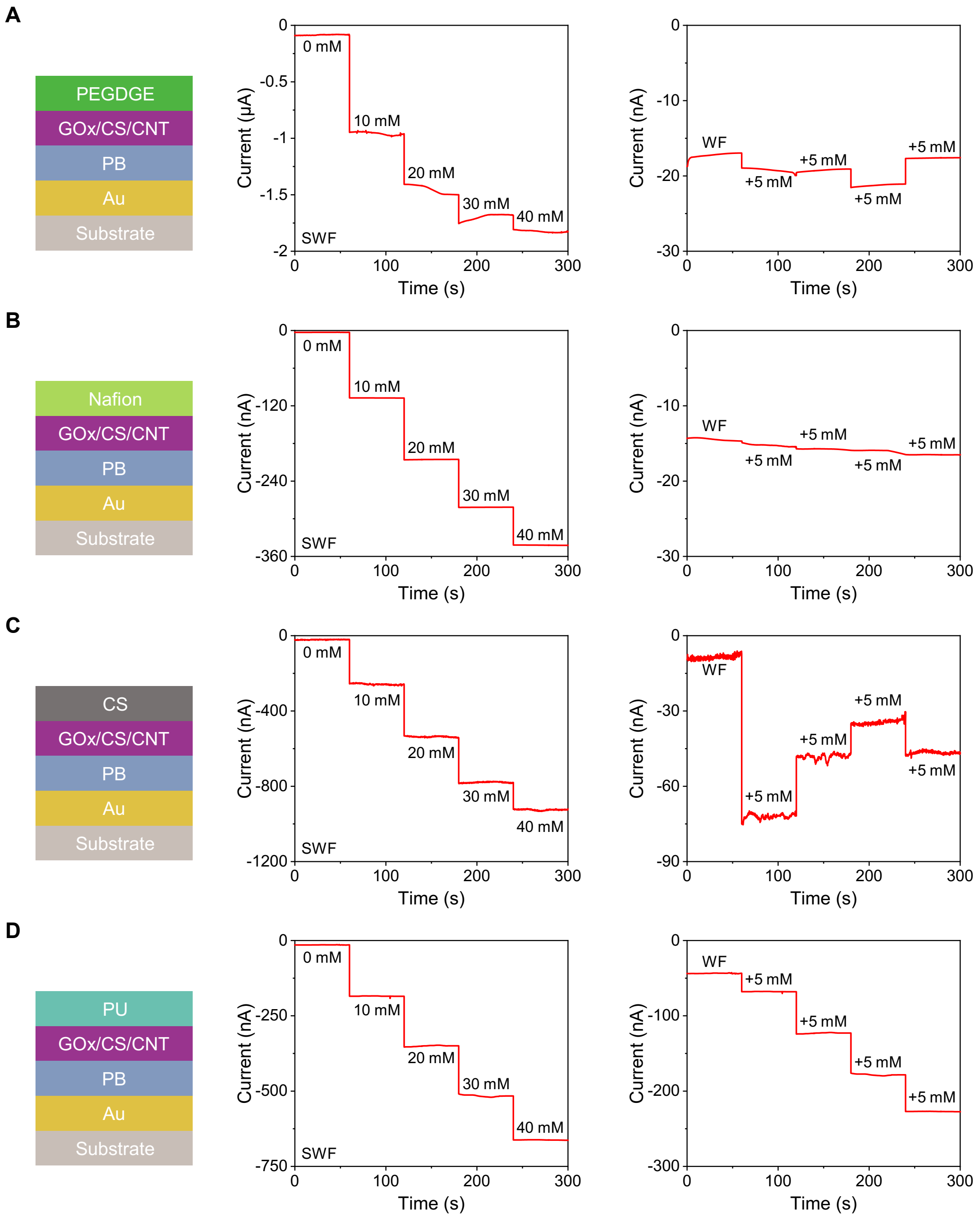

Fig. S9. The performance of enzymatic glucose sensors with different diffusion limit layers in SWF samples and WF samples collected from mice. (**A** to **D**) Schematic and performance of the GOx/CS/MWCNTs glucose sensors coated with poly(ethylene glycol) diglycidyl ether (PEGDGE) (**A**), Nafion (**B**), CS (**C**), and polyurethane (PU) (**D**) layers in SWF and WF. The WF samples were collected after 24 hours of fasting.

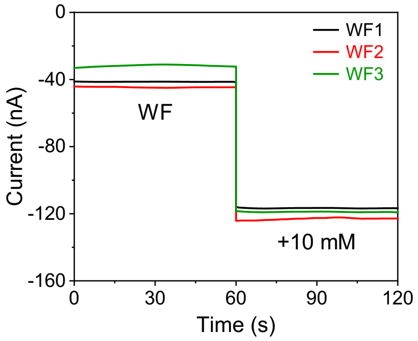

Fig. S10. Evaluation of the PU-coated glucose sensors in WF samples collected from three different mice. The WF samples were collected after 24 hours of fasting.

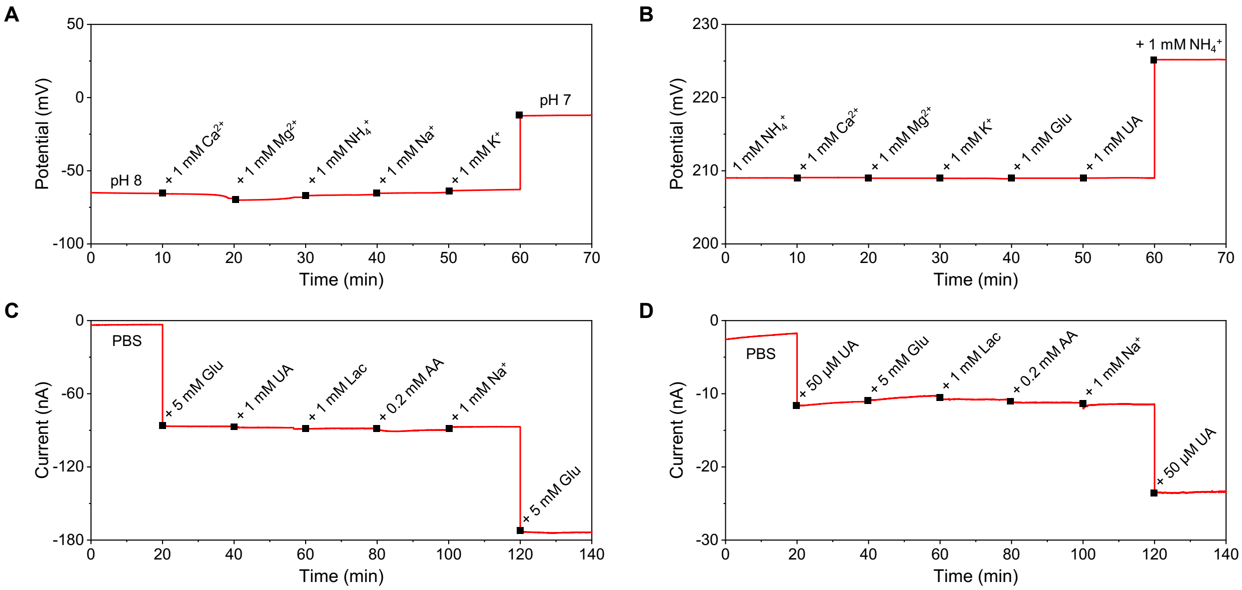

Fig. S11. Selectivity of the biosensors in SWF. (**A** and **B**) Selectivity of the pH (**A**) and NH_4^+ sensors against other ions in SWF. (**C** and **D**) Selectivity of the glucose (**C**) and UA (**D**) sensors against other metabolites and electrolytes in SWF. AA, ascorbic acid.

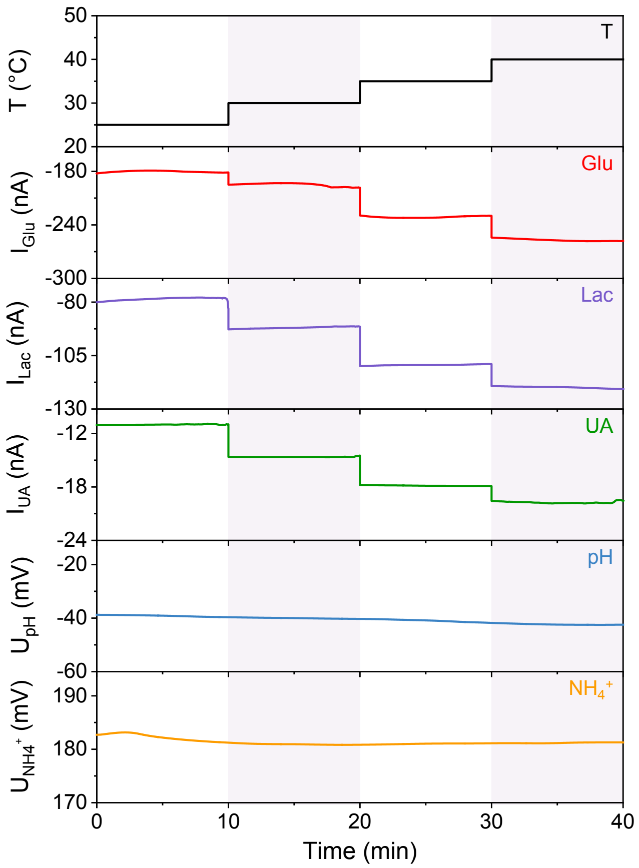

Fig. S12. The influence of temperature on the responses of multiplex biosensors in SWF. The SWF (pH 8.0) contains 10 mM glucose, 50 μM UA, 1 mM lactate, and 0.25 mM NH_4^+.

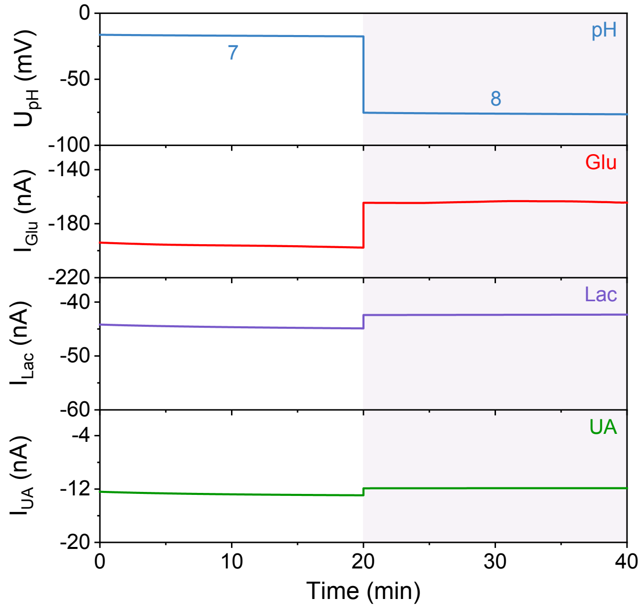

Fig. S13. The influence of pH on the responses of multiplex sensors in SWF. The SWF contains 10 mM glucose, 50 µM UA, 1 mM lactate, and 0.25 mM NH_4^+.

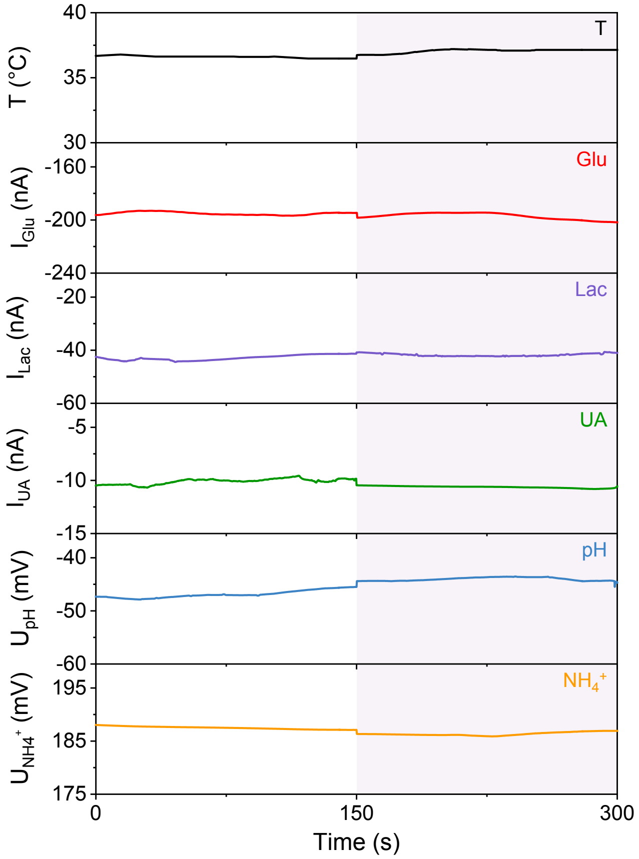

Fig. S14. The mechanical stability of the multiplex sensor array. Data was recorded before and after 100 cycles of repetitive mechanical bending (radius of curvature, 1 cm). The SWF (pH 8.0) contains 10 mM glucose, 50 µM UA, 1 mM lactate, and 0.25 mM NH_4^+.

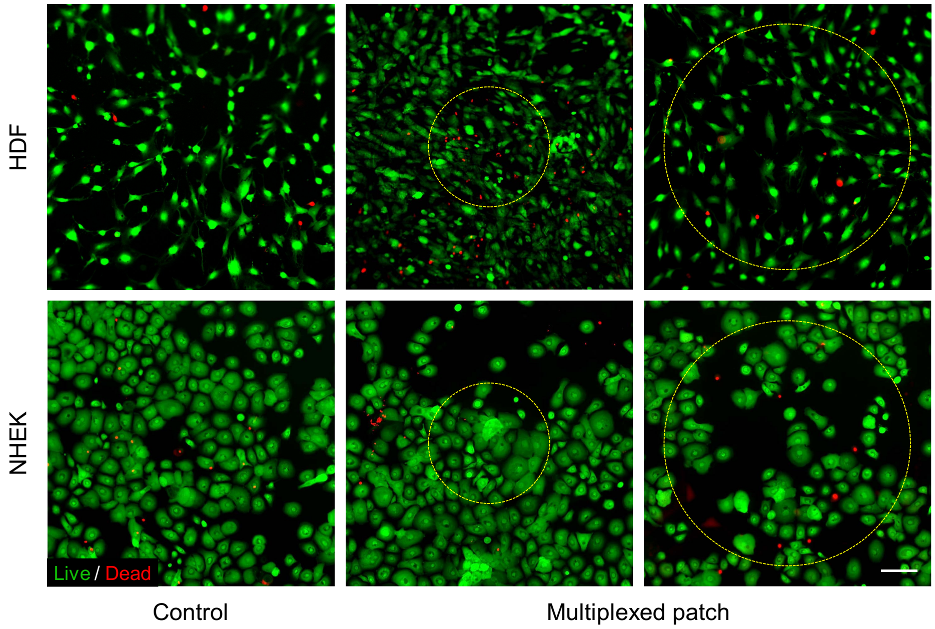

Fig. S15. *In vitro* cytocompatibility of the wearable patch. Representative live (green)/dead(red) images of human dermal fibroblasts (HDF) and normal human epidermal keratinocytes (NHEK) cells seeded on the multiplexed sensor array after 4-day culture. Scale bar, 200 µm.

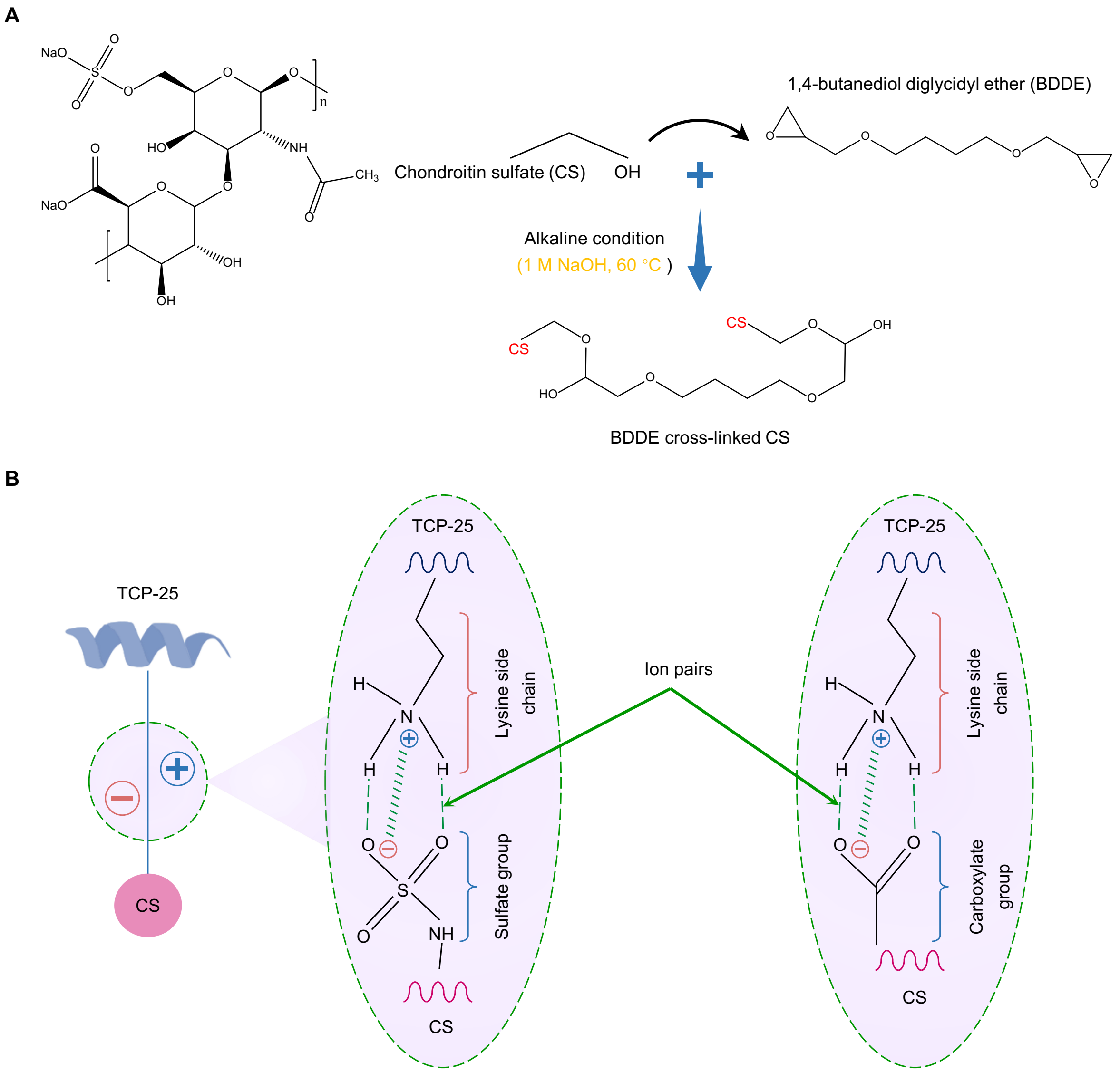

Fig. S16. Schematics of the electroactive hydrogel formation and drug loading process. (**A**) Schematic of chemical crosslinking process of chondroitin sulfate (CS) with 1,4-Butanediol diglycidyl ether (BDDE). (**B**) Schematic of the drug loading mechanism *via* the ion pair interactions between lysine groups in TCP-25 AMP and sulfate/carboxylate groups in CS.

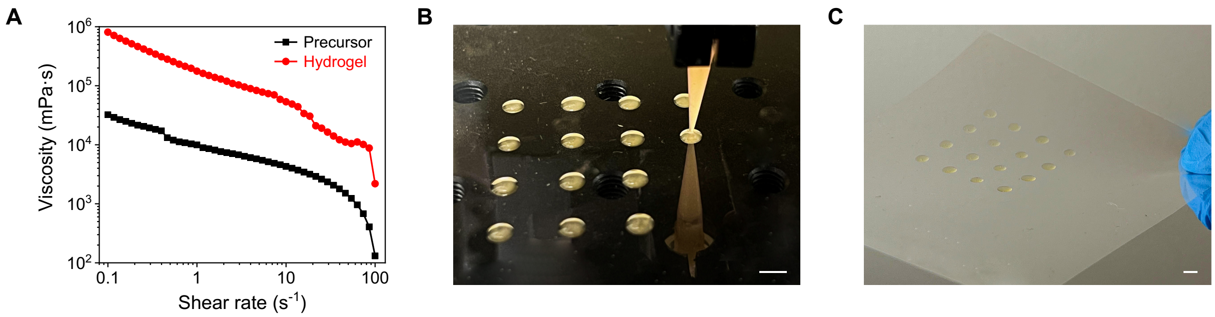

Fig. S17. Rheological properties and printability of electroactive hydrogel. (**A**) Dynamic viscosity of precursor and electroactive hydrogel at different shear rates. (**B** and **C**) Images of 3D printed electroactive hydrogels before (**B**) and after (**C**) crosslinking. Scale bars, 3 mm.

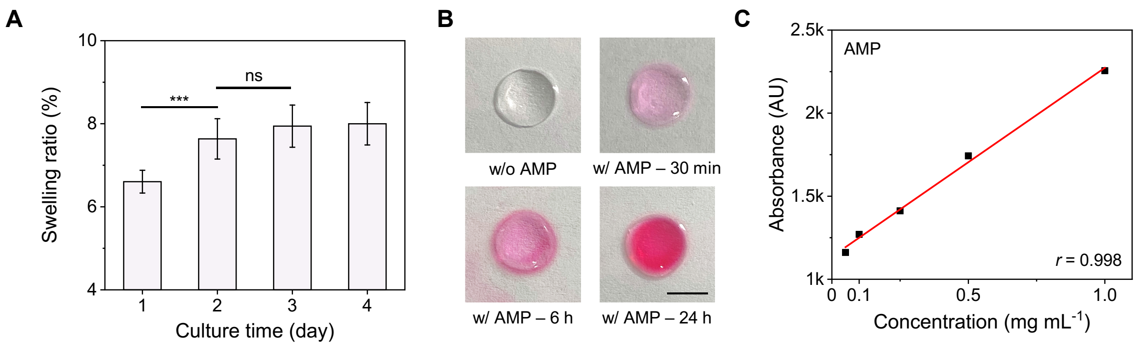

Fig. S18. Characterization of drug loading in the electroactive hydrogels. (**A**) Swelling ratios of electroactive hydrogel in DI water at 4˚C. Error bars represent the s.d. (**$p<0.01$, ns: not significant; n=3). (**B**) Photos illustrating the loading process of TCP-25 AMP-labeled with tetramethylrhodamine (TAMRA) into the electroactive hydrogel. Scale bars, 5 mm. (**C**) Calibration curve for the loading amount of the TAMRA labeled AAMP versus concentration used in the loading process.

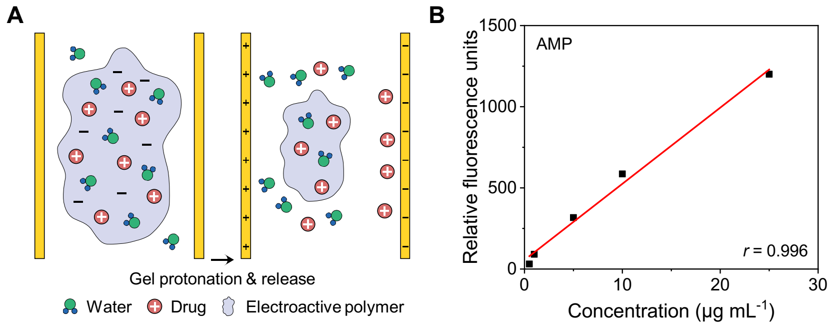

Fig. S19. Controlled drug release from the wearable patch under electrical field. (**A**) Schematic representation of the electrically accelerated release of positively-charged AMP drug molecules (red circles) from negatively-charged CS hydrogel. Under the applied electric field, the CS hydrogel undergoes protonation and shrinkage, as well as neutralization of the negative charge and consequently the release of the positively-charged AMP. (**B**) Calibration curve between the relative fluorescence signal and TAMRA-labeled AMP concentration to investigate the drug release.

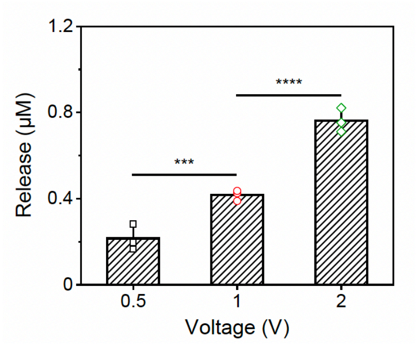

Fig. S20. Effect of applied potential on drug release from electroactive hydrogel. Error bars represent the s.d. (***$p < 0.001$, and ****$p < 0.0001$; n=3).

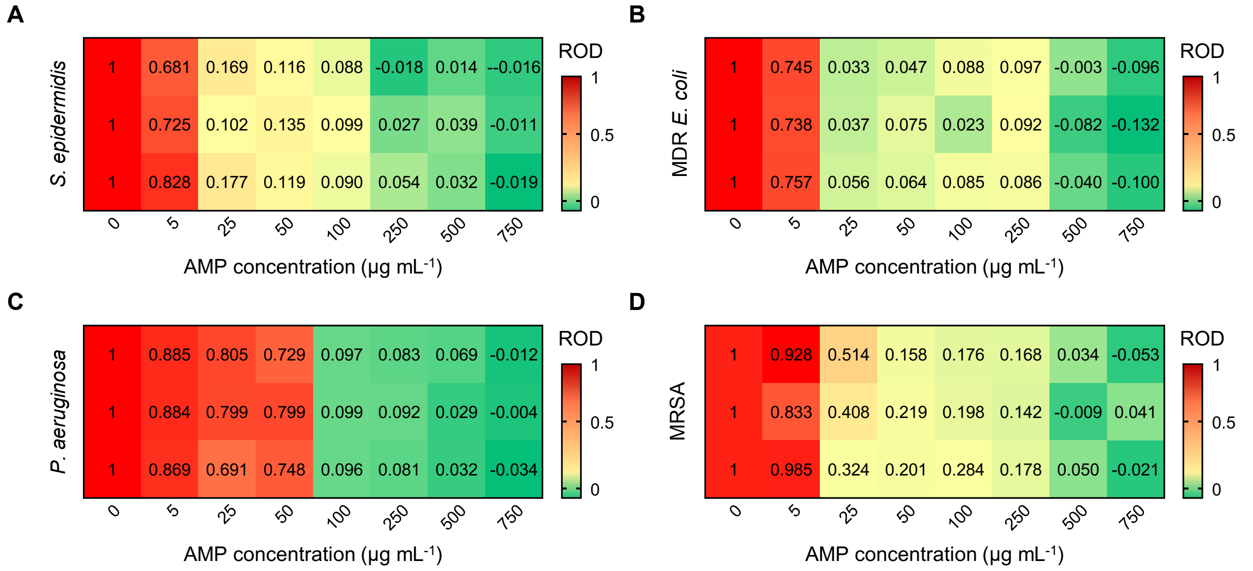

Fig. S21. Minimum inhibitory concentration assay for TCP-25 AMP against different pathogenic bacteria. (**A** to **D**) Relative optical density growth showing the minimum inhibitory concentrations for TCP-25 AMP against *S. epidermidis* (**A**), MDR *E. coli* (**B**), *P. aeruginosa* (**C**), MRSA (**D**). ROD, relative optical density.

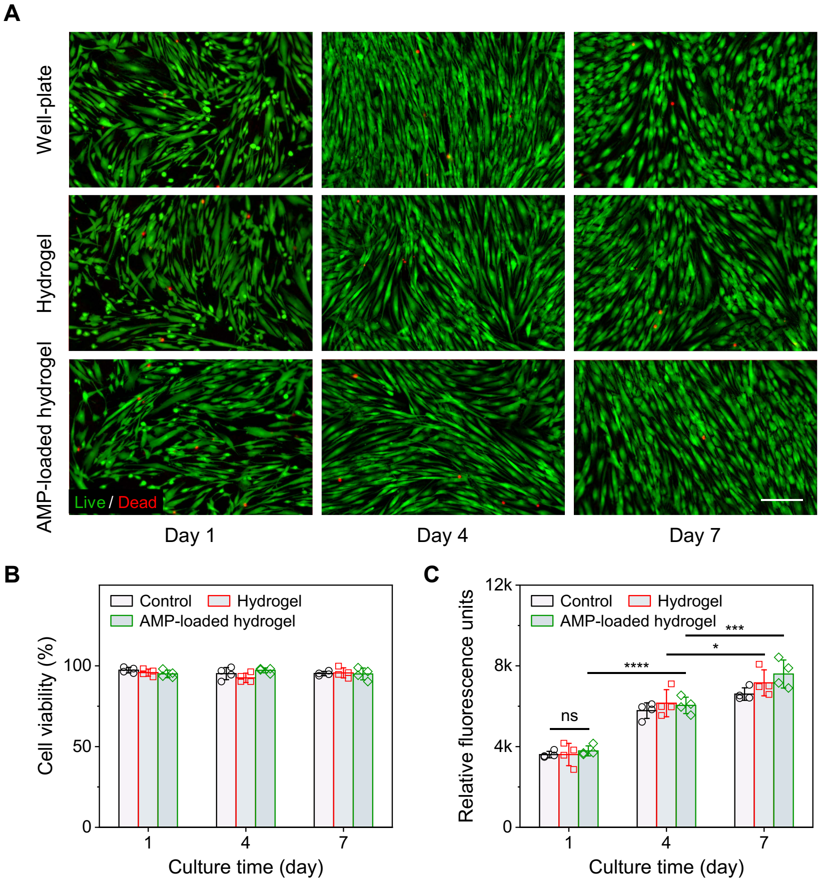

Fig. S22. ***In vitro*** **cytocompatibility of the electroactive hydrogel.** (**A** to **C**) *In vitro* cytocompatibility assessment of bare hydrogel and AMP-loaded hydrogel using live/dead staining assay (**A**,**B**) and metabolic activity analysis (**C**) for HDF cells seeded on the hydrogels after 1-day, 4-day, and 7-day culture. Scale bar, 100 µm. Error bars represent the s.d. (*$p < 0.05$, ***$p < 0.001$, and ****$p < 0.0001$; $n \geq 3$).

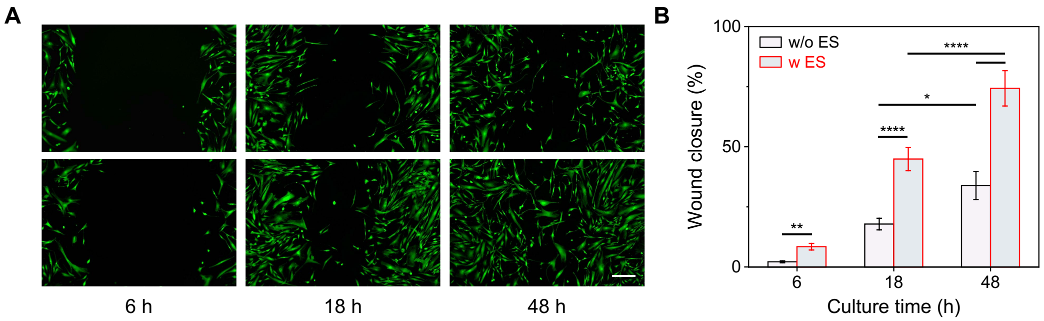

Fig. S23. Evaluation of electrical stimulation facilitated wound healing *in vitro*. (**A** and **B**) Fluorescence images (**A**) and quantitative wound closure analysis (**B**) show wound closure facilitated *via* electrical stimulation using an *in vitro* scratch wound healing model created with HDF cells. A pulsed voltage was applied in a perpendicular direction on a pair of parallel electrodes for electrical stimulation (1 V at 50 Hz, 0.01 s voltage on for each cycle). Scale bar, 500 µm. Error bars represent the s.d. (*$p < 0.05$, **$p < 0.01$, and ****$p < 0.0001$; n=3).

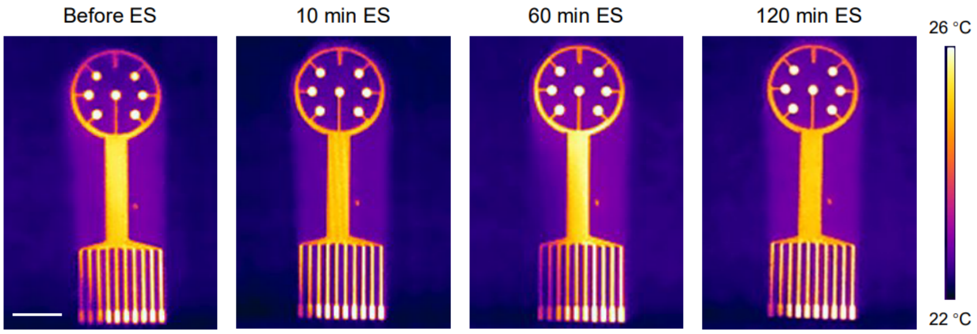

Fig. S24. Infrared photos of the patch temperature during the electrical stimulation. Scale bar, 5 mm.

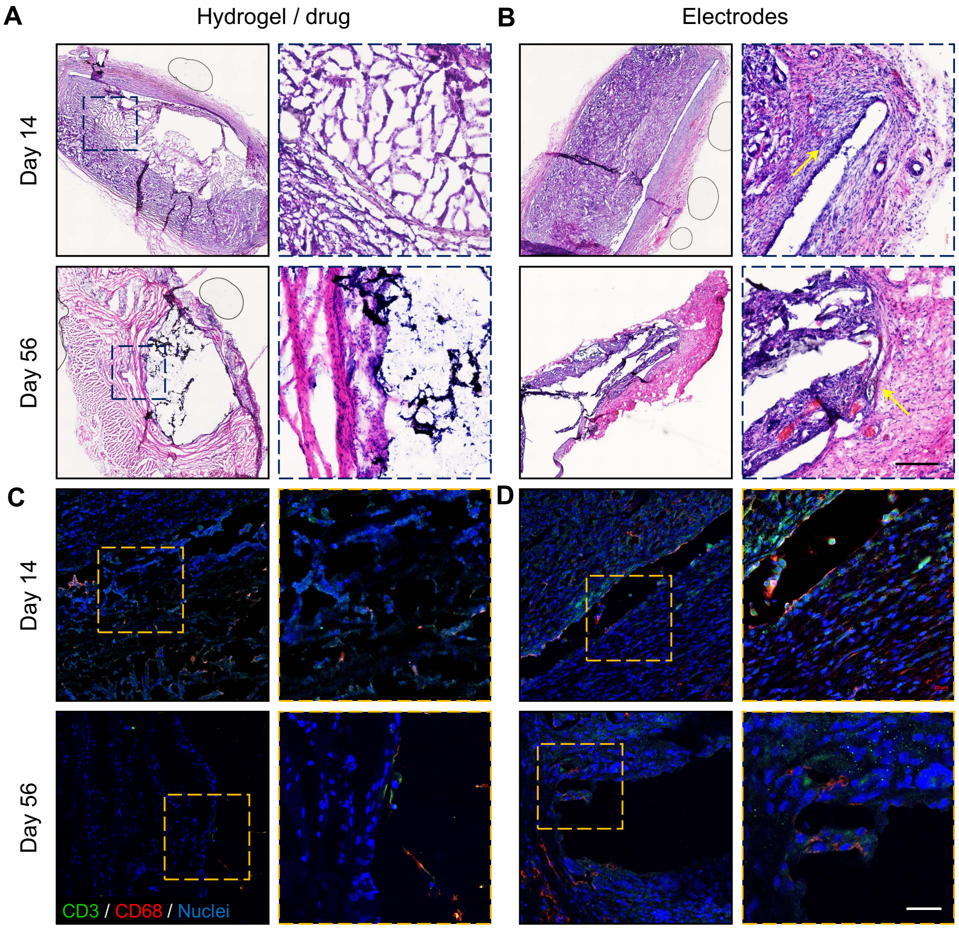

Fig. S25. *In vivo* biocompatibility of the wearable patch. (**A** and **B**) Hematoxylin and eosin (H&E) staining of AMP-loaded hydrogel (**A**) and wearable patch (**B**) with the surrounding tissue after 14 days and 56 days of implantation. Scale bar, 1 mm. (**C** and **D**) Fluorescent immunohistochemical analysis of subcutaneously implanted AMP-loaded hydrogel (**C**) and wearable patch (**D**) showing no significant infiltration at days 14 and 56. Green, red, and blue colors represent lymphocyte (CD3), macrophages (CD68), and cell nuclei, respectively. Scale bar, 100 µm.

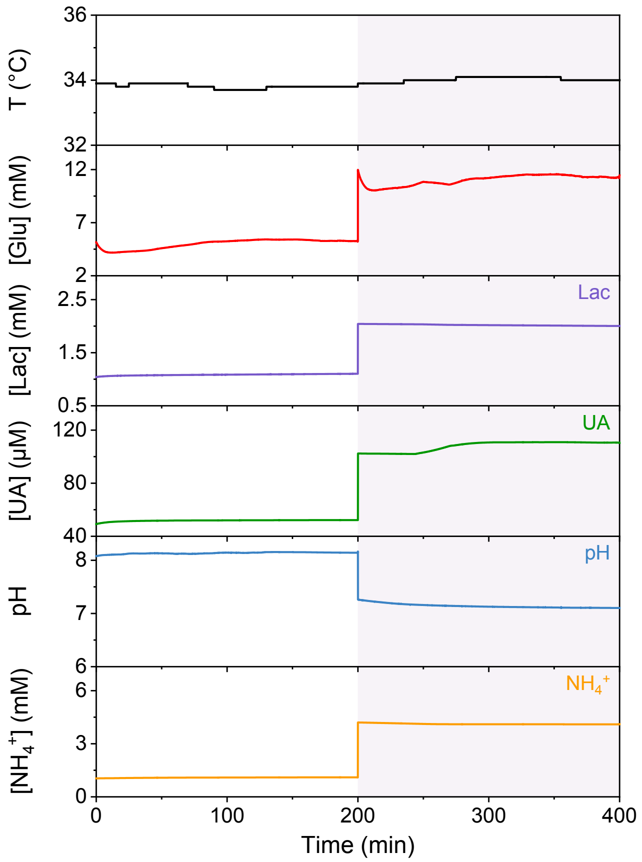

Fig. S26. Long term stability study of the wearable patch for multiplexed wound analysis. The study was performed firstly in SWF (pH 8) containing 5 mM glucose, 50 µM UA, 1 mM lactate, and 1 mM NH_4^+ for the first 200 min, and then in SWF (pH 7) containing 10 mM glucose, 100 µM UA, 2 mM lactate, and 4 mM NH_4^+ for the later the 200 min. The data for enzymatic sensors are calibrated based on pH influence.

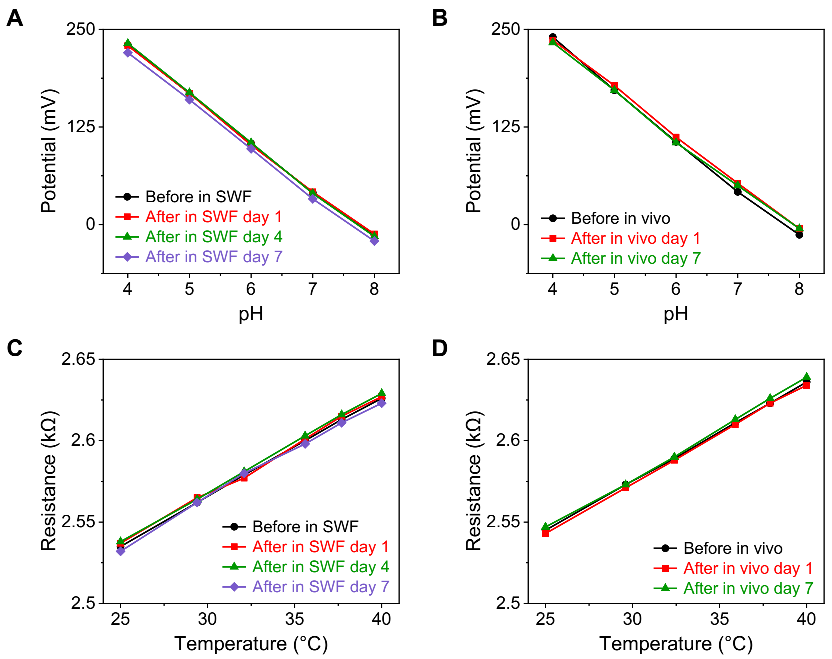

Fig. S27. Stability of pH and temperature sensors before and after incubation in SWF and *in vivo* experiments in rats. (**A** and **B**) Stability of pH sensors before and after incubation in SWF (**A**) and *in vivo* experiments in an infected wound model in ZDF diabetic rats (**B**). (**C** and **D**) Stability of temperature sensors before and after incubation in SWF (**C**) and *in vivo* experiments in an infected wound model in ZDF diabetic rats (**D**).

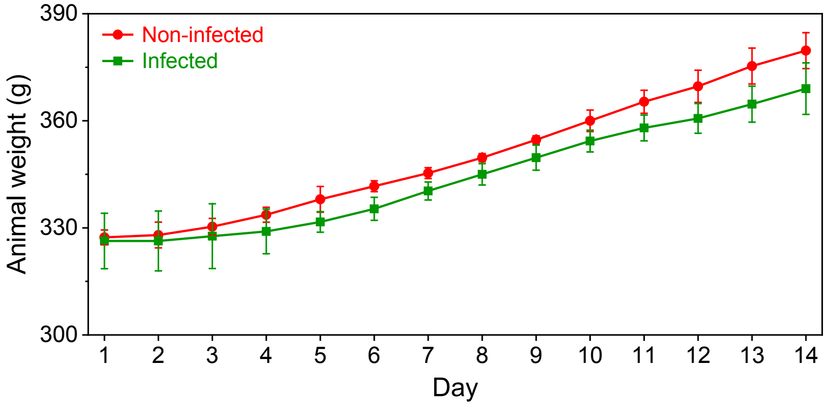

Fig. S28. Changes in weights of ZDF diabetic rats with non-infected and infected wounds. Error bars represent the s.d. from 3 rats.

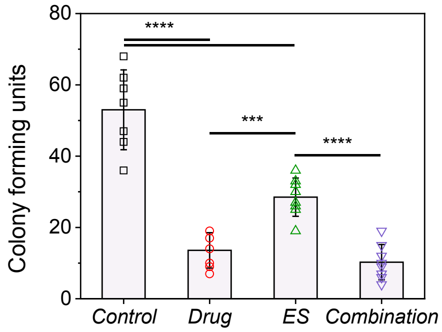

Fig. S29. ***In vivo* antimicrobial properties of wearable patch during chronic wound healing test in full-thickness infected wounds in ZDF diabetic rats.** The colony forming assay on bacteria samples isolated from control, drug, electrical stimulation (ES), and combination therapy group on day 3 post treatment. Error bars represent the s.d. (***p < 0.001, and ****p < 0.0001; n ≥ 3).

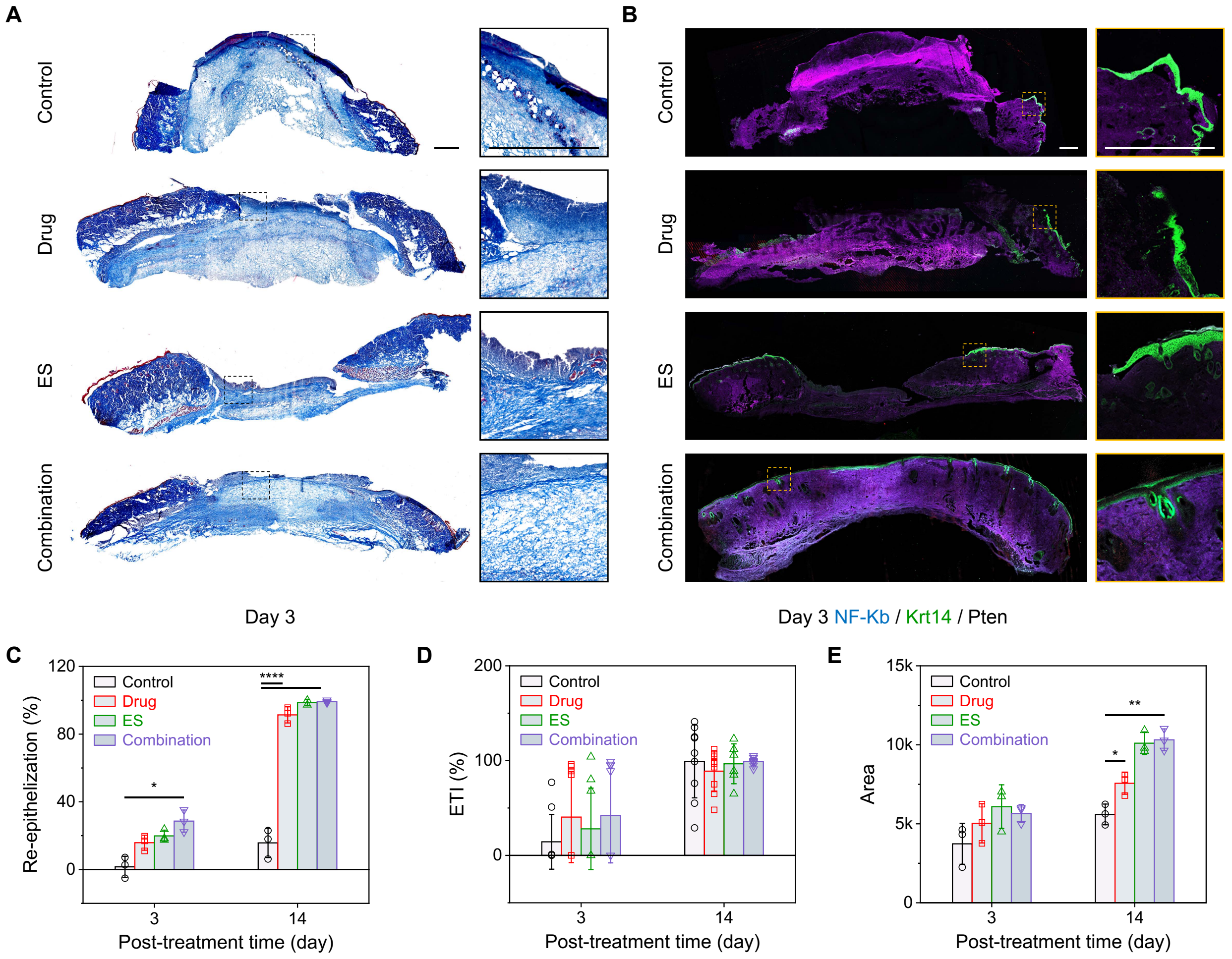

Fig. S30. *In vivo* evaluation of wearable patch-facilitated chronic wound healing in full-thickness infected wounds in ZDF diabetic rats. (**A**) Representative images of Masson's trichrome (MTC) stained sections of the full-thickness skin wounds after 3 days of treatment. Scale bar, 1 mm. (**B**) Representative immunofluorescent stained images for nuclear factor kappa B (NF-κB) (purple), keratin 14 (Krt14) (green), and phosphatase and tensin homolog (Pten) (red) 3 days after treatment. Scale bar, 1 mm. (**C** to **E**), Quantitative analysis of re-epithelization (%) (**C**) and epidermal thickness index (ETI) (**D**), and Pten marker based on immunofluorescent images (**E**). Error bars represent the s.d. (*$p<0.05$, **$p<0.01$, ****$p<0.0001$; $n=3$).

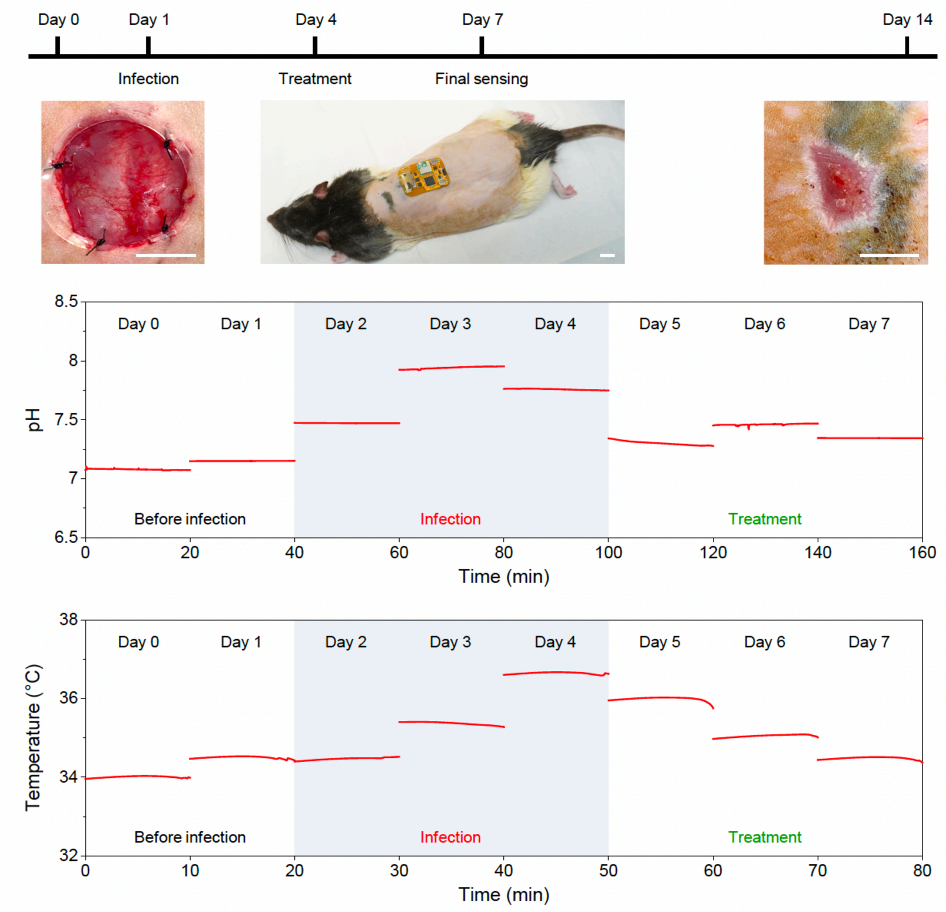

Fig. S31. Evaluation of the wearable system for multiplexed biosensing and the combination therapy on a diabetic rat. Infection and treatment were performed after the sensor recording on days 1 and 4, respectively. Scale bars, 1 cm.

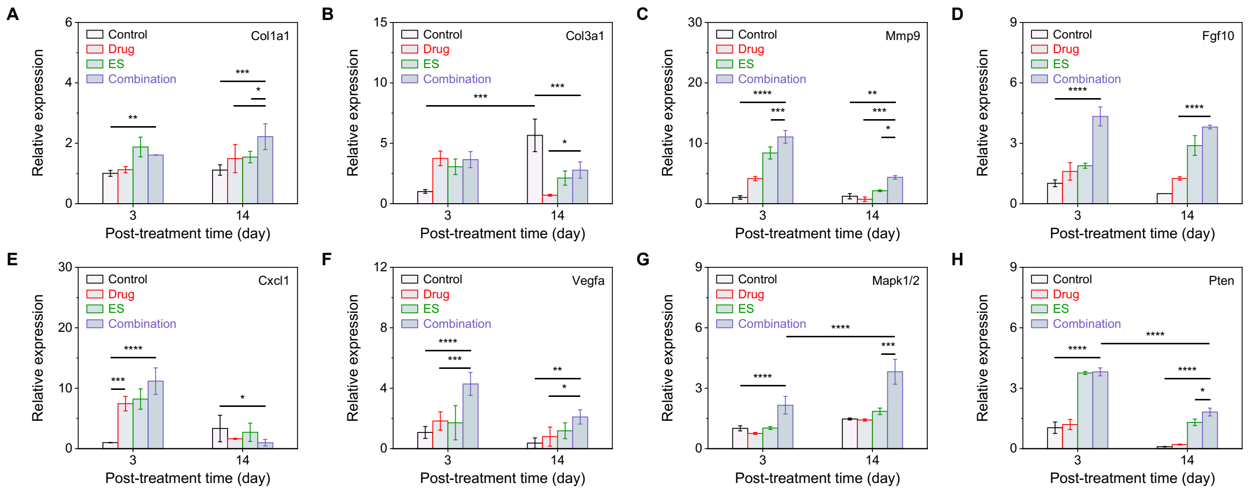

Fig. S32. Quantitative real-time polymerase chain reaction (qRT-PCR) analysis of a library of wound biomarkers. (**A** to **H**) Relative expression of Col1a1 (**A**), Col3a1 (**B**), Mmp9 (**C**), Fgf10 (**D**), Cxcl1 (**E**), Vegfa (**F**), Mapk1/2 (**G**), and Pten (**H**) genes after 3 and 14 days of treatment. Error bars represent the s.d. (*p<0.05, **p<0.01, ***p <0.001, ****p<0.0001; n=3).

Movie S1 (separate file). A rat moving freely with a wireless wearable device on the back.

REFERENCES AND NOTES

1. G. C. Gurtner, S. Werner, Y. Barrandon, M. T. Longaker, Wound repair and regeneration. *Nature* **453**, 314–321 (2008).

2. S. A. Eming, P. Martin, M. Tomic-Canic, Wound repair and regeneration: Mechanisms, signaling, and translation. *Sci. Transl. Med.* **6**, 265sr6 (2014).

3. B. K. Sun, Z. Siprashvili, P. A. Khavari, Advances in skin grafting and treatment of cutaneous wounds. *Science* **346**, 941–945 (2014).

4. C. E. Fife, M. J. Carter, Wound care outcomes and associated cost among patients treated in US outpatient wound centers: Data from the US wound registry. *Wounds* **24**, 10–17 (2012).

5. D. G. Armstrong, A. J. M. Boulton, S. A. Bus, Diabetic foot ulcers and their recurrence. *N. Engl. J. Med.* **376**, 2367–2375 (2017).

6. R. G. Frykberg, J. Banks, Challenges in the treatment of chronic wounds. *Adv. Wound Care* **4**, 560–582 (2015).

7. E. Eriksson, P. Y. Liu, G. S. Schultz, M. M. Martins-Green, R. Tanaka, D. Weir, L. J. Gould, D. G. Armstrong, G. W. Gibbons, R. Wolcott, O. O. Olutoye, R. S. Kirsner, G. C. Gurtner, Chronic wounds: Treatment consensus. *Wound Repair Regen.* **30**, 156–171 (2022).

8. J. M. A. Blair, M. A. Webber, A. J. Baylay, D. O. Ogbolu, L. J. V. Piddock, Molecular mechanisms of antibiotic resistance. *Nat. Rev. Microbiol.* **13**, 42–51 (2015).

9. G. Thakral, J. LaFontaine, B. Najafi, T. K. Talal, P. Kim, L. A. Lavery, Electrical stimulation to accelerate wound healing. *Diabet. Foot Ankle* **4**, 22081 (2013).

10. L. C. Kloth, Electrical stimulation technologies for wound healing. *Adv. Wound Care* **3**, 81–90 (2014).

11. S. Patel, A. Maheshwari, A. Chandra, Biomarkers for wound healing and their evaluation. *J. Wound Care* **25**, 46–55 (2016).

12. L. E. Lindley, O. Stojadinovic, I. Pastar, M. Tomic-Canic, Biology and biomarkers for wound healing. *Plast. Reconstr. Surg.* **138**, 18S–28S (2016).

13. K. F. Cutting, Wound exudate: Composition and functions. *Br. J. Community Nurs.* **8**, S4–S9 (2003).

14. R. A. Nery, B. S. Kahlow, T. L. Skare, F. I. Tabushi, A. do A. e Castro, Uric acid and tissue repair. *Arq. Bras. Cir. Dig.* **28**, 290–292 (2015).

15. S. Britland, O. Ross-Smith, H. Jamil, A. G. Smith, K. Vowden, P. Vowden, The lactate conundrum in wound healing: Clinical and experimental findings indicate the requirement for a rapid point-of-care diagnostic. *Biotechnol. Prog.* **28**, 917–924 (2012).

16. T. Hirsch, M. Spielmann, B. Zuhaili, T. Koehler, M. Fossum, H.-U. Steinau, F. Yao, L. Steinstraesser, A. B. Onderdonk, E. Eriksson, Enhanced susceptibility to infections in a diabetic wound healing model. *BMC Surg.* **8**, 5 (2008).

17. C. Xu, Y. Yang, W. Gao, Skin-interfaced sensors in digital medicine: From materials to applications. *Matter* **2**, 1414–1445 (2020).

18. T. R. Ray, J. Choi, A. J. Bandodkar, S. Krishnan, P. Gutruf, L. Tian, R. Ghaffari, J. A. Rogers, Bio-integrated wearable systems: A comprehensive review. *Chem. Rev.* **119**, 5461–5533 (2019).

19. J. Kim, A. S. Campbell, B. E.-F. de Ávila, J. Wang, Wearable biosensors for healthcare monitoring. *Nat. Biotechnol.* **37**, 389–406 (2019).

20. Y. Yang, W. Gao, Wearable and flexible electronics for continuous molecular monitoring. *Chem. Soc. Rev.* **48**, 1465–1491 (2019).

21. T. Someya, Z. Bao, G. G. Malliaras, The rise of plastic bioelectronics. *Nature* **540**, 379–385 (2016).

22. Z. Zhou, K. Chen, X. Li, S. Zhang, Y. Wu, Y. Zhou, K. Meng, C. Sun, Q. He, W. Fan, E. Fan, Z. Lin, X. Tan, W. Deng, J. Yang, J. Chen, Sign-to-speech translation using machine-learning-assisted stretchable sensor arrays. *Nat. Electron.* **3**, 571–578 (2020).

23. Y. Zhou, X. Zhao, J. Xu, Y. Fang, G. Chen, Y. Song, S. Li, J. Chen, Giant magnetoelastic effect in soft systems for bioelectronics. *Nat. Mater.* **20**, 1670–1676 (2021).

24. W. Gao, S. Emaminejad, H. Y. Y. Nyein, S. Challa, K. Chen, A. Peck, H. M. Fahad, H. Ota, H. Shiraki, D. Kiriya, D.-H. Lien, G. A. Brooks, R. W. Davis, A. Javey, Fully integrated wearable sensor arrays for multiplexed in situ perspiration analysis. *Nature* **529**, 509–514 (2016).

25. T. R. Ray, M. Ivanovic, P. M. Curtis, D. Franklin, K. Guventurk, W. J. Jeang, J. Chafetz, H. Gaertner, G. Young, S. Rebollo, J. B. Model, S. P. Lee, J. Ciraldo, J. T. Reeder, A. Hourlier-Fargette, A. J. Bandodkar, J. Choi, A. J. Aranyosi, R. Ghaffari, S. A. McColley, S. Haymond, J. A. Rogers, Soft, skin-interfaced sweat stickers for cystic fibrosis diagnosis and management. *Sci. Transl. Med.* **13**, eabd8109 (2021).

26. H. Lee, T. K. Choi, Y. B. Lee, H. R. Cho, R. Ghaffari, L. Wang, H. J. Choi, T. D. Chung, N. Lu, T. Hyeon, S. H. Choi, D.-H. Kim, A graphene-based electrochemical device with thermoresponsive microneedles for diabetes monitoring and therapy. *Nat. Nanotechnol.* **11**, 566–572 (2016).

27. A. Koh, D. Kang, Y. Xue, S. Lee, R. M. Pielak, J. Kim, T. Hwang, S. Min, A. Banks, P. Bastien, M. C. Manco, L. Wang, K. R. Ammann, K.-I. Jang, P. Won, S. Han, R. Ghaffari, U. Paik, M. J. Slepian, G. Balooch, Y. Huang, J. A. Rogers, A soft, wearable microfluidic device for the capture, storage, and colorimetric sensing of sweat. *Sci. Transl. Med.* **8**, 366ra165 (2016).

28. F. Tehrani, H. Teymourian, B. Wuerstle, J. Kavner, R. Patel, A. Furmidge, R. Aghavali, H. Hosseini-Toudeshki, C. Brown, F. Zhang, K. Mahato, Z. Li, A. Barfidokht, L. Yin, P. Warren, N. Huang, Z. Patel, P. P. Mercier, J. Wang, An integrated wearable microneedle array for the continuous monitoring of multiple biomarkers in interstitial fluid. *Nat. Biomed. Eng.* **6**, 1214–1224 (2022).

29. J. R. Sempionatto, M. Lin, L. Yin, E. De la paz, K. Pei, T. Sonsa-ard, A. N. de Loyola Silva, A. A. Khorshed, F. Zhang, N. Tostado, S. Xu, J. Wang, An epidermal patch for the simultaneous monitoring of haemodynamic and metabolic biomarkers. *Nat. Biomed. Eng.* **5**, 737–748 (2021).

30. C. Wang, X. Li, H. Hu, L. Zhang, Z. Huang, M. Lin, Z. Zhang, Z. Yin, B. Huang, H. Gong, S. Bhaskaran, Y. Gu, M. Makihata, Y. Guo, Y. Lei, Y. Chen, C. Wang, Y. Li, T. Zhang, Z. Chen, A. P.

Pisano, L. Zhang, Q. Zhou, S. Xu, Monitoring of the central blood pressure waveform via a conformal ultrasonic device. *Nat. Biomed. Eng.* **2**, 687–695 (2018).

31. C. Wang, E. Shirzaei Sani, W. Gao, Wearable bioelectronics for chronic wound management. *Adv. Funct. Mater.* **32**, 2111022 (2022).

32. Y. Jiang, A. A. Trotsyuk, S. Niu, D. Henn, K. Chen, C.-C. Shih, M. R. Larson, A. M. Mermin-Bunnell, S. Mittal, J.-C. Lai, A. Saberi, E. Beard, S. Jing, D. Zhong, S. R. Steele, K. Sun, T. Jain, E. Zhao, C. R. Neimeth, W. G. Viana, J. Tang, D. Sivaraj, J. Padmanabhan, M. Rodrigues, D. P. Perrault, A. Chattopadhyay, Z. N. Maan, M. C. Leeolou, C. A. Bonham, S. H. Kwon, H. C. Kussie, K. S. Fischer, G. Gurusankar, K. Liang, K. Zhang, R. Nag, M. P. Snyder, M. Januszyk, G. C. Gurtner, Z. Bao, Wireless closed-loop smart bandage for chronic wound management and accelerated tissue regeneration. *bioRxiv*, 2022.01.16.476432 (2022).

33. R. Dong, B. Guo, Smart wound dressings for wound healing. *Nano Today* **41**, 101290 (2021).

34. Z. Xiong, S. Achavananthadith, S. Lian, L. E. Madden, Z. X. Ong, W. Chua, V. Kalidasan, Z. Li, Z. Liu, P. Singh, H. Yang, S. P. Heussler, S. M. P. Kalaiselvi, M. B. H. Breese, H. Yao, Y. Gao, K. Sanmugam, B. C. K. Tee, P.-Y. Chen, W. Loke, C. T. Lim, G. S. H. Chiang, B. Y. Tan, H. Li, D. L. Becker, J. S. Ho, A wireless and battery-free wound infection sensor based on DNA hydrogel. *Sci. Adv.* **7**, eabj1617 (2021).

35. V. Kalidasan, X. Yang, Z. Xiong, R. R. Li, H. Yao, H. Godaba, S. Obuobi, P. Singh, X. Guan, X. Tian, S. A. Kurt, Z. Li, D. Mukherjee, R. Rajarethinam, C. S. Chong, J.-W. Wang, P. L. R. Ee, W. Loke, B. C. K. Tee, J. Ouyang, C. J. Charles, J. S. Ho, Wirelessly operated bioelectronic sutures for the monitoring of deep surgical wounds. *Nat. Biomed. Eng.* **5**, 1217–1227 (2021).

36. Y. Gao, D. T. Nguyen, T. Yeo, S. B. Lim, W. X. Tan, L. E. Madden, L. Jin, J. Y. K. Long, F. A. B. Aloweni, Y. J. A. Liew, M. L. L. Tan, S. Y. Ang, S. D. Maniya, I. Abdelwahab, K. P. Loh, C.-H. Chen, D. L. Becker, D. Leavesley, J. S. Ho, C. T. Lim, A flexible multiplexed immunosensor for point-of-care in situ wound monitoring. *Sci. Adv.* **7**, eabg9614 (2021).

37. P. Kassal, J. Kim, R. Kumar, W. R. de Araujo, I. M. Steinberg, M. D. Steinberg, J. Wang, Smart bandage with wireless connectivity for uric acid biosensing as an indicator of wound status. *Electrochem. Commun.* **56**, 6–10 (2015).

38. M. Puthia, M. Butrym, J. Petrlova, A.-C. Strömdahl, M. Å. Andersson, S. Kjellström, A. Schmidtchen, A dual-action peptide-containing hydrogel targets wound infection and inflammation. *Sci. Transl. Med.* **12**, eaax6601 (2020).

39. T. Tanaka, I. Nishio, S.-T. Sun, S. Ueno-Nishio, Collapse of gels in an electric field. *Science* **218**, 467–469 (1982).

40. C. M. Proctor, A. Slézia, A. Kaszas, A. Ghestem, I. del Agua, A.-M. Pappa, C. Bernard, A. Williamson, G. G. Malliaras, Electrophoretic drug delivery for seizure control. *Sci. Adv.* **4**, eaau1291 (2018).

41. B. Song, Y. Gu, J. Pu, B. Reid, Z. Zhao, M. Zhao, Application of direct current electric fields to cells and tissues in vitro and modulation of wound electric field in vivo. *Nat. Protoc.* **2**, 1479–1489 (2007).

42. M. Zhao, B. Song, J. Pu, T. Wada, B. Reid, G. Tai, F. Wang, A. Guo, P. Walczysko, Y. Gu, T. Sasaki, A. Suzuki, J. V. Forrester, H. R. Bourne, P. N. Devreotes, C. D. McCaig, J. M. Penninger, Electrical signals control wound healing through phosphatidylinositol-3-OH kinase-γ and PTEN. *Nature* **442**, 457–460 (2006).

43. D. J. Cohen, W. James Nelson, M. M. Maharbiz, Galvanotactic control of collective cell migration in epithelial monolayers. *Nat. Mater.* **13**, 409–417 (2014).

44. A. Chanmugam, D. Langemo, K. Thomason, J. Haan, E. A. Altenburger, A. Tippett, L. Henderson, T. A. Zortman, Relative temperature maximum in wound infection and inflammation as compared with a control subject using long-wave infrared thermography. *Adv. Skin Wound Care* **30**, 406–414 (2017).

45. M. L. Fernandez, Z. Upton, H. Edwards, K. Finlayson, G. K. Shooter, Elevated uric acid correlates with wound severity. *Int. Wound J.* **9**, 139–149 (2012).

46. G. Tegl, D. Schiffer, E. Sigl, A. Heinzle, G. M. Guebitz, Biomarkers for infection: Enzymes, microbes, and metabolites. *Appl. Microbiol. Biotechnol.* **99**, 4595–4614 (2015).

47. M. Digirolamo, F. D. Newby, J. Lovejoy, Lactate production in adipose tissue; a regulated function with extra-adipose implications. *FASEB J.* **6**, 2405–2412 (1992).

48. I. Sakakibara, T. Fujino, M. Ishii, T. Tanaka, T. Shimosawa, S. Miura, W. Zhang, Y. Tokutake, J. Yamamoto, M. Awano, S. Iwasaki, T. Motoike, M. Okamura, T. Inagaki, K. Kita, O. Ezaki, M. Naito, T. Kuwaki, S. Chohnan, T. T. Yamamoto, R. E. Hammer, T. Kodama, M. Yanagisawa, J. Sakai, Fasting-induced hypothermia and reduced energy production in mice lacking acetyl-CoA synthetase 2. *Cell Metab.* **9**, 191–202 (2009).

49. D. R. Griffin, M. M. Archang, C.-H. Kuan, W. M. Weaver, J. S. Weinstein, A. C. Feng, A. Ruccia, E. Sideris, V. Ragkousis, J. Koh, M. V. Plikus, D. Di Carlo, T. Segura, P. O. Scumpia, Activating an adaptive immune response from a hydrogel scaffold imparts regenerative wound healing. *Nat. Mater.* **20**, 560–569 (2021).

50. Y. R. Park, Md. T. Sultan, H. J. Park, J. M. Lee, H. W. Ju, O. J. Lee, D. J. Lee, D. L. Kaplan, C. H. Park, NF-κB signaling is key in the wound healing processes of silk fibroin. *Acta Biomater.* **67**, 183–195 (2018).

51. A. Leask, Potential therapeutic targets for cardiac Fibrosis. *Circ. Res.* **106**, 1675–1680 (2010).

52. C. Profyris, C. Tziotzios, I. Do Vale, Cutaneous scarring: Pathophysiology, molecular mechanisms, and scar reduction therapeutics Part I. The molecular basis of scar formation. *J. Am. Acad. Dermatol.* **66**, 1–10; quiz 11–2 (2012).

53. I. Gerling, C. Nejman, N. K. Chatterjee, Effect of coxsackievirus B4 infection in mice on expression of 64,000-Mr autoantigen and glucose sensitivity of islets before development of hyperglycemia. *Diabetes* **37**, 1419–1425 (1988).

54. T. M. Simone, C. E. Higgins, R.-P. Czekay, B. K. Law, S. P. Higgins, J. Archambeault, S. M. Kutz, P. J. Higgins, SERPINE1: A molecular switch in the proliferation-migration dichotomy in wound-"activated" keratinocytes. *Adv. Wound Care* **3**, 281–290 (2014).

55. H. Yu, R. Jove, The STATs of cancer--new molecular targets come of age. *Nat. Rev. Cancer* **4**, 97–105 (2004).

56. M. Wang, Y. Yang, J. Min, Y. Song, J. Tu, D. Mukasa, C. Ye, C. Xu, N. Heflin, J. S. McCune, T. K. Hsiai, Z. Li, W. Gao, A wearable electrochemical biosensor for the monitoring of metabolites and nutrients. *Nat. Biomed. Eng* **6**, 1225–1235 (2022).

57. Y. Yang, Y. Song, X. Bo, J. Min, O. S. Pak, L. Zhu, M. Wang, J. Tu, A. Kogan, H. Zhang, T. K. Hsiai, Z. Li, W. Gao, A laser-engraved wearable sensor for sensitive detection of uric acid and tyrosine in sweat. *Nat. Biotechnol.* **38**, 217–224 (2020).

58. A. J. Bandodkar, P. Gutruf, J. Choi, K. Lee, Y. Sekine, J. T. Reeder, W. J. Jeang, A. J. Aranyosi, S. P. Lee, J. B. Model, R. Ghaffari, C. J. Su, J. P. Leshock, T. Ray, A. Verrillo, K. Thomas, V. Krishnamurthi, S. Han, J. Kim, S. Krishnan, T. Hang, J. A. Rogers, Battery-free, skin-interfaced microfluidic/electronic systems for simultaneous electrochemical, colorimetric, and volumetric analysis of sweat. *Sci. Adv.* **5**, eaav3294 (2019).

59. C. A. P. Quinn, R. E. Connor, A. Heller, Biocompatible, glucose-permeable hydrogel for in situ coating of implantable biosensors. *Biomaterials* **18**, 1665–1670 (1997).

60. Y. Yu, J. Nassar, C. Xu, J. Min, Y. Yang, A. Dai, R. Doshi, A. Huang, Y. Song, R. Gehlhar, A. D. Ames, W. Gao, Biofuel-powered soft electronic skin with multiplexed and wireless sensing for human-machine interfaces. *Sci. Robot.* **5**, eaaz7946 (2020).

61. Y. Song, J. Min, Y. Yu, H. Wang, Y. Yang, H. Zhang, W. Gao, Wireless battery-free wearable sweat sensor powered by human motion. *Sci. Adv.* **6**, eaay9842 (2020).

62. S. Park, S. W. Heo, W. Lee, D. Inoue, Z. Jiang, K. Yu, H. Jinno, D. Hashizume, M. Sekino, T. Yokota, K. Fukuda, K. Tajima, T. Someya, Self-powered ultra-flexible electronics via nano-grating-patterned organic photovoltaics. *Nature* **561**, 516–521 (2018).

63. J. Chen, Y. Huang, N. Zhang, H. Zou, R. Liu, C. Tao, X. Fan, Z. L. Wang, Micro-cable structured textile for simultaneously harvesting solar and mechanical energy. *Nat. Energy* **1**, 16138 (2016).

64. S. S. Veidal, E. Vassiliadis, N. Barascuk, C. Zhang, T. Segovia-Silvestre, L. Klickstein, M. R. Larsen, P. Qvist, C. Christiansen, B. Vainer, M. A. Karsdal, Matrix metalloproteinase-9-mediated type III collagen degradation as a novel serological biochemical marker for liver fibrogenesis. *Liver Int.* **30**, 1293–1304 (2010).

65. K. Kessenbrock, V. Plaks, Z. Werb, Matrix metalloproteinases: Regulators of the tumor microenvironment. *Cell* **141**, 52–67 (2010).

66. Z. I. Elbialy, D. H. Assar, A. Abdelnaby, S. A. Asa, E. Y. Abdelhiee, S. S. Ibrahim, M. M. Abdel-Daim, R. Almeer, A. Atiba, Healing potential of Spirulina platensis for skin wounds by modulating bFGF, VEGF, TGF-ß1 and α-SMA genes expression targeting angiogenesis and scar tissue formation in the rat model. *Biomed. Pharmacother.* **137**, 111349 (2021).

67. M. Presta, G. Andrés, D. Leali, P. Dell'Era, R. Ronca, Inflammatory cells and chemokines sustain FGF2-induced angiogenesis. *Eur. Cytokine Netw.* **20**, 39–50 (2009).

68. D. Wang, J. Sai, A. Richmond, Cell surface heparan sulfate participates in CXCL1-induced signaling. *Biochemistry* **42**, 1071–1077 (2003).

69. P. Bao, A. Kodra, M. Tomic-Canic, M. S. Golinko, H. P. Ehrlich, H. Brem, The role of vascular endothelial growth factor in wound healing. *J. Surg. Res.* **153**, 347–358 (2009).
